# Vaccine value profile for *Neisseria gonorrhoeae*

**DOI:** 10.1016/j.vaccine.2023.01.053

**Published:** 2023-12-13

**Authors:** Yiming Lyu, Annabelle Choong, Eric P.F. Chow, Kate L. Seib, Helen S. Marshall, Magnus Unemo, Alex de Voux, Bing Wang, Angelica E. Miranda, Sami L. Gottlieb, Maeve B. Mello, Teodora Wi, Rachel Baggaley, Caroline Marshall, Laith J. Abu-Raddad, Winston E. Abara, Xiang-Sheng Chen, Jason J. Ong

**Affiliations:** a University of Melbourne, Parkville, Victoria 3010, Australia; b Central Clinical School, Faculty of Medicine, Nursing and Health Sciences, Monash University, 99 Commercial Road, Melbourne, Victoria 3004, Australia; c Melbourne Sexual Health Centre, Alfred Health, 580 Swanston Street, Melbourne, Victoria 3053, Australia; d Centre for Epidemiology and Biostatistics, Melbourne School of Population and Global Health, The University of Melbourne, 207 Bouverie Street, Melbourne, Victoria 3053, Australia; e Institute for Glycomics, Griffith University, Gold Coast, Queensland 4222, Australia; f The University of Adelaide, Adelaide, South Australia 5005, Australia; g Women’s and Children’s Health Network, North Adelaide, South Australia, Australia; h WHO CC for Gonorrhoea and Other STIs, Örebro University, Örebro, Sweden; i Institute for Global Health, University College London (UCL), London, UK; j Division of Epidemiology and Biostatistics, Faculty of Health Sciences, University of Cape Town, Rondebosch, Cape Town 7700, South Africa; k Department of Social Medicine, Universidade Federal do Espirito Santo, Av. Fernando Ferrari, 514 - Goiabeiras, Vitória – ES 29075-910, Brazil; l Department of Sexual and Reproductive Health and Research, World Health Organization, Av. Appia 20, 1211 Genève, Switzerland; m Global HIV, Hepatitis and STI Programmes, World Health Organization, Av. Appia 20, 1211 Genève, Switzerland; n Department of Immunization, Vaccines and Biologicals, World Health Organization, Av. Appia 20, 1211 Genève, Switzerland; o Weill Cornell Medicine-Qatar, Qatar Foundation – Education City, Box 24144, Doha, Qatar; p Centers for Disease Control and Prevention, 1600 Clifton Road, Atlanta, GA 30329, USA; q National Center for STD Control of China CDC, 12 Jiangwangmiao Street, Nanjing 210042, China

**Keywords:** *Neisseria gonorrhoeae*, Gonorrhoea, Sexually transmitted infection, Infertility, Vaccine, Value profile, OMV MenB vaccines

## Abstract

*Neisseria gonorrhoeae* infection (gonorrhoea) is a global public health challenge, causing substantial sexual and reproductive health consequences, such as infertility, pregnancy complications and increased acquisition or transmission of HIV. There is an urgency to controlling gonorrhoea because of increasing antimicrobial resistance to ceftriaxone, the last remaining treatment option, and the potential for gonorrhoea to become untreatable. No licensed gonococcal vaccine is available. Mounting observational evidence suggests that *N. meningitidis* serogroup B outer membrane vesicle-based vaccines may induce cross-protection against *N. gonorrhoeae* (estimated 30%–40% effectiveness using the 4CMenB vaccine). Clinical trials to determine the efficacy of the 4CMenB vaccine against *N. gonorrhoeae* are underway, as are Phase 1/2 studies of a new gonococcal-specific vaccine candidate. Ultimately, a gonococcal vaccine must be accessible, affordable and equitably dispensed, given that those most affected by gonorrhoea are also those who may be most disadvantaged in our societies, and most cases are in less-resourced settings.

This vaccine value profile (VVP) provides a high level, holistic assessment of the current data to inform the potential public health, economic and societal value of pipeline vaccines. This was developed by a working group of subject matter experts from academia, non-profit organizations, public private partnerships and multi-lateral organizations. All contributors have extensive expertise on various elements of the *N. gonorrhoeae* VVP and collectively aimed to identify current research and knowledge gaps. The VVP was developed using published data obtained from peer-reviewed journals or reports.

## Summary

The development of this Vaccine Value Profile has been commissioned by WHO’s Immunization, Vaccines and Biologicals (IVB) department on the recommendation of IVB’s Product Development for Vaccines Advisory Committee, to an independent contractor. All authors are independent subject matter experts and the authors alone are responsible for the views expressed in this manuscript.

## 1. Introduction

### 1.1. The global public health need for a vaccine

*Neisseria gonorrhoeae* infection (gonorrhoea) is a global public health challenge. There are an estimated 82 million new gonorrhoea cases among adults each year, with the highest disability-adjusted life years (DALYs) in low- and middle-income countries (LMICs), particularly in sub-Saharan Africa, Southeast Asia and Oceania [1,2]. There is urgency in controlling gonorrhoea because of increasing antimicrobial resistance (AMR) and the potential for gonorrhoea to become untreatable [3]. Untreated gonorrhoea can lead to a wide range of sexual and reproductive health consequences, including infertility, adverse pregnancy outcomes, and elevated risk of HIV acquisition and transmission. Globally, there has been variable success in controlling gonorrhoea (through public health campaigns, treatment efforts, sexual health education, behavioural counselling, and encouraging consistent condom use). The incidence is rising in high-income countries, primarily among key populations. In LMICs, lack of access to testing has resulted in no significant change in incidence. Furthermore, the pipeline of new antimicrobials for the treatment of gonorrhoea is scarce.

No licensed gonococcal vaccine is available. However, there is renewed interest in developing a gonococcal vaccine after reports of decreased rates of gonorrhoea by 31 % amongst those vaccinated with a strain-specific outer membrane vesicle (OMV) meningococcal serogroup B vaccine (MeNZB) in a retrospective case-control study of 14,730 sexual health clinic attendees in New Zealand [4]. Further evidence from two studies from the United States and Australia confirmed that the 4CMenB vaccine (Bexsero, which includes MeNZB’s OMV, but also contains additional antigens) could lower the acquisition of gonorrhoea by 30–40 % [5,6]. The World Health Organization (WHO) released preferred product characteristics (PPCs) for gonococcal vaccines in 2021 [7,8], and called for vaccine development to achieve the global health sector strategy for sexually transmitted infections (STIs) target to reduce gonorrhoea incidence by 90 % by 2030 [9].

### 1.2. Current methods of surveillance, diagnosis, prevention, and treatment

#### 1.2.1. AMR surveillance

High-quality and representative gonococcal AMR data are imperative to monitor AMR trends, identify emerging AMR, and inform refinements of global, international and national clinical management guidelines and public health policies. The WHO Global Gonococcal Antimicrobial Surveillance Programme (GASP) has documented the emergence and spread of AMR in gonorrhoea globally since 1992. The GASP is a worldwide laboratory network coordinated by focal points and regional coordinating centres. In partnership with its WHO regional office, each designated regional focal point collates data on antimicrobial susceptibility patterns in gonorrhoea in participating countries. Surveillance data should be collected – including breakpoints for AMR, frequency of collection, number of isolates, and interpretation of local AMR data. Further guidance for surveillance of AMR in *N. gonorrhoeae* is available from WHO [67]. A WHO Enhanced GASP (EGASP) has also been established and is currently expanded internationally. The WHO EGASP collects quality-assured and standardised gonococcal AMR data linked to key clinical and epidemiological variables [68].

#### 1.2.2. Laboratory diagnosis

Primarily, diagnosis of gonococcal infections is made with nucleic acid amplification testing (NAAT), but culture and Gram stain are also options [69]. NAAT is the preferred method because of its high sensitivity and specificity, but it is expensive and requires more sophisticated laboratory infrastructure and technical staff to provide accurate results, making it relatively inaccessible in many LMICs. NAAT does not provide information on antimicrobial susceptibility, so culture should be conducted in those diagnosed with gonococcal infections by NAAT to guide management and monitor patterns of AMR [70].

Culturing *N. gonorrhoeae* requires good samples collected at the point of care, proper transportation of samples and appropriate laboratory culture techniques. The process can be complicated because *N. gonorrhoeae* viability is difficult to maintain during transport. Culture offers a cheaper alternative to NAAT but has lower sensitivity. Gram stain microscopy can diagnose symptomatic gonococcal infection in male urethra but has poor sensitivity and frequently suboptimal specificity for samples collected in the cervix, rectum, and oropharynx [70].

In most LMICs, laboratory testing for gonorrhoea is not possible due to the lack of resources. Instead, *N. gonorrhoeae* infection is confirmed based on clinical presentation. This approach works relatively well for men as they are more likely to present with symptoms of gonococcal urethritis, but most gonococcal infections in women as well as oropharyngeal and rectal infections are asymptomatic and are therefore at risk of being undiagnosed [70]. The lack of access to antimicrobial susceptibility testing, the low sensitivity and the poor positive predictive value (PPV) of syndromic management of gonorrhoea are concerning, as this can result in lack of treatment, incorrect antimicrobial treatment as well as overuse of antimicrobial therapies, further fueling the current crisis of AMR [71].

#### 1.2.3. Prevention

Sexual health promotion remains the major prevention method for *N. gonorrhoeae* infection; this includes public health campaigns, sexual health education, behavioural counselling, and consistent condom use. A review article published in the Bulletin of the World Health Organization presented multiple studies that demonstrated a significant reduction in the incidence of gonorrhoea in association with condom use [72]. Though the effectiveness of safe sex education and promotion of condom use has contributed to limiting *N. gonorrhoeae* infection, in the last two decades, adherence to condom use during sex has waned, coinciding with the expansion of other HIV prevention methods such as treatment as prevention (TasP) and pre-exposure prophylaxis (PrEP) [73]. Prophylactic use of antibiotics (e.g. doxycycline) may reduce incidence of bacterial STIs, however, this is not currently recommended pending the results of ongoing clinical trials (Tables 1–4).

#### 1.2.4. Treatment

The current recommended first-line empirical antimicrobial therapy in most countries is a combined antimicrobial regimen of intramuscular ceftriaxone 250–1000 mg and oral azithromycin 1–2 g [70,74]. Some countries such as the UK and the US have moved away from recommending this dual therapy to just a monotherapy of ceftriaxone 1000 mg and 500 mg, respectively [75,76]. This therapy can clear most gonococcal infections but is under significant threat of treatment failure since the first occurrence of unsuccessfully cleared pharyngeal gonorrhoea with recommended ceftriaxone plus azithromycin dual therapy in 2016. Only after treatment with an increased dosage of ceftriaxone and azithromycin did the pharyngeal swab return negative [77]. In 2018, the UK and Australia reported the first gonorrhoea case resistant to ceftriaxone and showing high-level resistance to azithromycin [78]. In 2022, a new *N. gonorrhoeae* strain with resistance to ceftriaxone combined with high-level azithromycin resistance caused a failure to treat a urethral gonorrhoea case in Austria [79], raising further concerns of gonorrhoea becoming an untreatable infection.

Since the emergence of ceftriaxone-resistant gonococcal strains, gonorrhoea has developed resistance against all available antimicrobial monotherapies. Effective antimicrobials are key to gonorrhoea control, and until any vaccine becomes available, new antimicrobials must be developed and tested for treating gonorrhoea. A few new antimicrobials are already studied for treating *N. gonorrhoeae* infection, such as gepotidacin and zoliflodacin. Nevertheless, further research is still required [54].

### 1.3. Summary of knowledge and research gaps in epidemiology, potential indirect public health impact and economic burden

The meeting report from a WHO global consultation on the public health value and PPCs of gonococcal vaccines outlined the knowledge gaps, data and research needs for developing gonococcal vaccines [8]. See Section 10 below for details on these research gaps.

## 2. Potential target populations and delivery strategies

The WHO PPC document outlines preferences and key considerations related to target populations for gonococcal vaccines [7]. The choice of the target population(s), e.g., broad-based vaccination of young people and/or targeted vaccination of specific populations at higher risk for gonococcal infection, may vary across different settings, and will depend on factors such as:

- gonococcal epidemiology.
- vaccine efficacy in those with prior infection.
- duration of vaccine protection.
- cost-effectiveness analyses.
- programmatic considerations.

In settings where target populations for the MenB vaccine already include young people, such as those entering university or military recruits, vaccinating with an OMV-based MenB vaccine to also prevent gonococcal infection could be relatively straightforward [7]. However, the uptake of existing MenB vaccines is low globally. Expanding the indication of the existing MenB vaccine to include the prevention of gonococcal infection could improve the cost-effectiveness of the vaccine and expand its use in more countries.

## 3. *Neisseria gonorrhoeae* and its consideration as a public health priority by global, regional or country stakeholders

The potential for recent increases in gonococcal AMR to compromise gonorrhoea control and worsen sexual and reproductive health outcomes has led stakeholders at the global, regional, and country level to consider *Neisseria gonorrhoeae* a public health priority. Global strategies related to both STIs and AMR have recognized gonorrhoea as one of the prioritized pathogens requiring immediate action for control [3,7,93–95], and this has been mirrored in regional and national action plans [96,97–99,100, 101–105]. In addition, several global vaccine-related initiatives reinforce the importance of gonococcal vaccine development as a public health priority, as do efforts related to the role of vaccines in the fight against AMR [100,101,104,105].

## 4. Existing guidance on preferences/preferred product attributes for vaccines against *Neisseria gonorrhoeae*

WHO has developed PPCs for gonococcal vaccines through a global consultative process [7]. Table 4 is reproduced from the WHO PPCs with permission. This table outlines the preferred characteristics and notes related to “ideal” gonococcal vaccines, namely, those specifically formulated to optimize efficacy against gonococcal infection and related adverse SRH outcomes, and includes additional notes for MenB vaccines with potential cross-protection against gonococcal infection.

## 5. Vaccine development

There is no protective immunity to natural infection with *N. gonorrhoeae*, and reinfections are common. No immunologic surrogates or correlates of protection against *N. gonorrhoeae* are known. However, observational studies of OMV-based *N. meningitidis* serogroup B (MenB) vaccines have demonstrated likely cross-protection against *N. gonorrhoeae*, with population-level reductions in gonococcal infection incidence as measured by NAATs [4–6]. Table 5 outlines considerations related to the feasibility of gonococcal vaccine development, and Table 6 summarizes the status of ongoing clinical trials of 4CMenB (Bexsero) and of the OMV-based *Neisseria gonorrhoeae* generalized modules for membrane antigens (GMMA) (NgG) investigational vaccine to prevent gonorrhoea. Other approaches in pre-clinical trials use purified protein subunit; natural and genetically modified OMVs from *N. gonorrhoeae* or *N. meningitidis*; or lipooligosaccharide epitope vaccines [34,108,109] (see Tables 7–10).

### 5.1. Probability of technical and regulatory success (PTRS)

See Table 5

### 5.2. Overview of the clinical trials of vaccine candidates

**Fig. 1.**
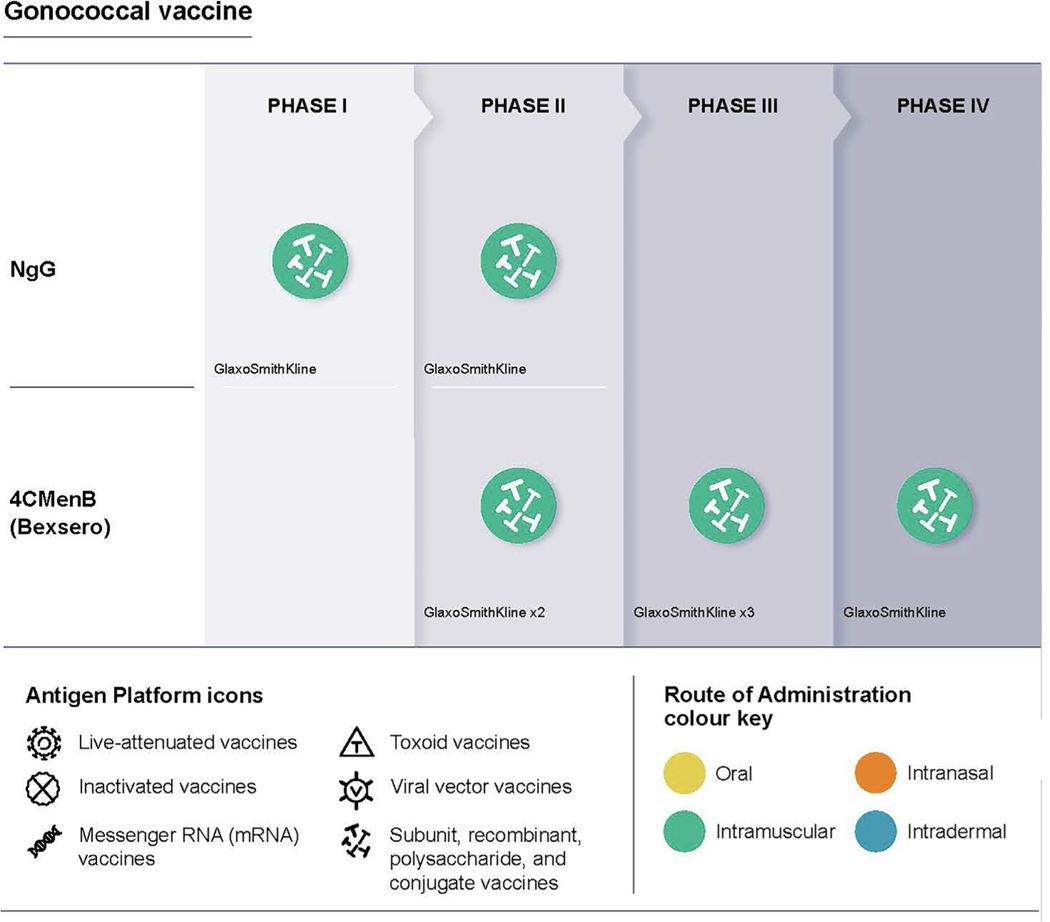
Overview of candidate vaccines for gonococcal infection in clinical trials.

See Table 6 and Fig. 1.

## 6. Health impact of a vaccine on the burden of disease and transmission

There is mounting evidence for the moderate effectiveness of the outer membrane vesicle-based vaccine, 4CMenB, in preventing gonorrhoea in vaccinated young people. Although this effect is consistent across several studies and is estimated at between 30 and 40 % using more robust methodologies such as matched case-control studies, the observational nature of these studies does not allow a causative effect to be substantiated. The randomised control trials (RCT) listed in Table 6 will provide direct data on the efficacy of 4CMenB and NgG against gonorrhoea. The impact of 4CMenB on gonorrhoea where co-infection occurs is conflicting, with some studies reporting lower effectiveness and others showing no difference. Long-term protection requires further evaluation as some studies suggest protection against gonorrhoea may wane after 3–4 years. Modelling studies indicate that even a low-efficacy vaccine against gonorrhoea, even as low as 20 %, could significantly reduce disease incidence and burden (Table 7). As women are more affected by the complications of gonococcal infection such as PID and infertility, any reduction in disease will improve women’s health globally.

## 7. Social and/or economic impact of a vaccine

Studies evaluating the social and/or economic impact of a gonococcal vaccine are limited (Table 7). Economic evaluation models in the US context demonstrated that a gonococcal vaccine would be needed to prevent rising costs of emerging AMR [131], and would be cost-saving [126].

## 8. Policy considerations and financing

At the global level, a WHO policy recommendation by the Strategic Advisory Group of Experts (SAGE) for immunization and WHO prequalification of gonococcal vaccines would be first steps for consideration of financing by Gavi, the Vaccine Alliance for low-income countries. Gavi evaluates new vaccines for financing in Gavi-eligible countries through its Vaccine Investment Strategy (VIS). The VIS process provides a transparent mechanism for assessing vaccine products using several criteria, including health and economic impact, the contribution of equity and social protection, feasibility and implementation costs. To date, the WHO Product Development for Vaccines Advisory Committee (PDVAC) has endorsed PPCs for gonococcal vaccines which are now published. PPCs describe vaccine attributes that would optimize impact, and outline critical data needs that are expected to facilitate policy decision-making. Key factors influencing the likelihood of global policy recommendations and financing include a better understanding of the global burden of disease due to gonorrhoea and the status of gonococcal AMR.

The greatest burden of gonorrhoea is in LMICs; however, given limited resources, Gavi-eligible countries will likely require Gavi financing to support the introduction of gonococcal vaccines. For LMICs that are not Gavi-eligible, policymakers need to make decisions on gonococcal vaccines based on the local epidemiologic context and the potential impact and cost-effectiveness of gonococcal vaccines. The same is true for HICs: despite greater resources for new vaccine introduction, the burden of gonococcal infection and disease is typically lower in general populations and mostly focused in smaller populations at higher risk. A WHO policy recommendation will be important for non-Gavi-eligible LMICs, but decisions will be informed by the country’s National Advisory Committees on Immunization. Generation of the data and evidence that will be needed for policymaking (at the national and global level) and Gavi financing can accelerate the pathway to vaccine introduction and use, in countries and target populations with the greatest need.

Of note, the policy and financing considerations for a standalone gonococcal vaccine will differ from those for MenB vaccines with some efficacy against gonorrhoea. An additional indication for an existing licensed/prequalified vaccine will simplify policy decision-making and likely improve cost-effectiveness given the potential impact on two conditions. However, the epidemiologic overlap between the two conditions in different settings, overlap in target populations, and cost and cost-effectiveness considerations will guide policy and financing decisions.

## 9. Access and implementation feasibility

Various aspects of access and implementation feasibility will differ for standalone gonococcal vaccines compared with MenB vaccines with potential cross-protection against gonococcal infection, as well as according to target populations (e.g., broad-based vaccination of adolescents versus targeted vaccination of individuals at higher risk) in different settings.

## 10. Research gaps

A WHO global consultation on the public health value and PPCs of gonococcal vaccines, held in January 2019, brought together a multidisciplinary, international group of experts to assess the current evidence base surrounding potential health, economic, and societal value of gonococcal vaccines, their likely acceptance and use, and preferred product considerations. The findings of this meeting were summarized and published [8]. Table 11, reproduced with permission from this meeting report, outlines the knowledge gaps, data and research needs for developing gonococcal vaccines. For references, please see the original meeting report.

## 11. Conclusion

The case for developing a vaccine for *N. gonorrhoeae* has become increasingly stronger, given an imminent threat of untreatable gonorrhoea with rapidly rising AMR. This potentially preventable STI affects an estimated 82 million people yearly [1], causing a substantial disease burden and economic costs. *N. gonorrhoeae* is a high-priority pathogen for addressing AMR by WHO and several other regional and national public health agencies and ministries of health.

Existing control measures have proved insufficient. In recent years, adherence to condom use during sex has been weaning off, coinciding with the expansion of HIV prevention methods such as TasP and PrEP for HIV infection [73]. Further, the stigma associated with STIs, the costs of accessing quality-assured molecular diagnostics associated with low testing coverage among priority populations, reliance on syndromic management leading to over-consumption of antibiotics, and inconsistent availability of antimicrobial susceptibility testing means that gonorrhoea is underdiagnosed and inadequately managed, particularly in LMICs. Screening and treating asymptomatic gonococcal infection would be ideal. However, even if affordable, feasible tests are made available globally, increasing AMR threatens the ability to use screening and treatment as a control measure.

Vaccination will be essential to control this pathogen and reach the WHO Global STI Strategy targets to reduce gonorrhoea incidence by 90 % by 2030 [9]. Humans can develop humoral and cellular responses following infections; however, these do not induce long-lasting immunity and protect against re-infection. Vaccine-induced immunity would need to be distinct from immune responses elicited through natural infection. Mounting evidence suggests that *N. meningitidis* serogroup B OMV-based vaccine may induce cross-protection against *N. gonorrhoeae* (30 %−40 % efficacy using the 4CMenB vaccinations), and clinical trials are underway to determine the efficacy of the 4CMenB vaccine against *N. gonorrhoeae*, along with Phase 1/2 studies of a new gonococcal-specific vaccine candidate. Population-based [4–6] and modelling studies [82,126–128] already demonstrate the potential to reduce infection burden and transmission, even if a vaccine is only moderately effective (as low as 20 %). Economic studies predict significant cost-savings and reductions in disease burden with scaling up gonococcal vaccination [126,131]. Integrating gonococcal vaccines with current vaccine delivery infrastructure for key target groups (e.g. young people through school-based programs) or opportunistic vaccination for specific populations at higher risk (e.g. MSM attending sexual health services), provides possibilities for implementation and scale-up.

Ultimately, a gonococcal vaccine must be affordable and equitably dispensed, given that those most affected by gonorrhoea are also those who may be most disadvantaged in our societies, and most cases are in less-resourced settings. To accelerate vaccine development and implementation, there are ongoing research priorities related to obtaining better epidemiologic data regarding *N. gonorrhoeae* infection, disease, AMR and natural history; modelling gonococcal infection, disease, AMR, economic burden and theoretical vaccine impact and cost-effectiveness, particularly from LMICs; advancing basic science, translational, immunobiologic, and clinical research; and encouraging investment and planning for policy and implementation decisions in advance.

## Supplementary Material

Supplementary 1

Supplementary 2

## Figures and Tables

**Table 1:** Summary of epidemiology of gonococcal infection and potential indirect public health impact.

Feature	Summary and evidence

*Epidemiology*	

Reservoir	• Humans are the only reservoir for the obligate human pathogen, *Neisseria gonorrhoeae.* Following sexual contact, *N. gonorrhoeae* can infect the mucosa of the urogenital tract, rectum, oropharynx and eyes. • *N. gonorrhoeae* can be transmitted vertically to the neonate during childbirth. • Asymptomatic infections with *N. gonorrhoeae* are common, particularly oropharyngeal, anorectal and endocervical infections. These asymptomatic infections are a significant reservoir of transmissible bacteria in the population [7,10–12].

Populations at higher risk	Men who have sex with men (MSM) • Reported gonococcal infections among MSM are up to 42 times higher than the estimated rate among men who have sex with women only • The estimated probability of transmission during condomless anal sex (urethral-to-rectal) is approximately 84% compared to penile-to-vaginal and vaginal-to-penile transmission, which is approximately 50% and 20% per sex act, respectively • Condom use for oral sex is rare, and condom use for anal sex has declined • Homophobia, discriminatory legislation and policies (e.g. the criminalisation of same-sex relationships), and stigma and discrimination in the health sector limit the access to and uptake of sexual health services in many countries Transgender and gender-diverse people • Risk varies within different subgroups within the transgender and gender-diverse community • Prevalence of gonorrhoea in the settings measured ranged from 2.1% to 19.1% in transgender women and 0% to 10.5% in transgender men. • Discriminatory laws and policies, criminalization and prosecution, high levels of violence, stigma and discrimination and lack of gender-inclusive and affirming care have been demonstrated to be major barriers to appropriate, safe and effective sexual healthcare services in most countries Sex workers (SW) and their clients • SW and their clients encompass a group of individuals of diverse genders and sexual orientations exposed to several societal and socioeconomic risk factors for acquiring gonorrhoea • Risk factors for the acquisition and transmission of infection for SW include: − Clients’ unwillingness to use condoms − Lack of access to condoms and low condom use with stable sexual partners − Financial hardship to access health services, including STI testing and treatment − Institutionalized discrimination, including in the health sector − Coercion, sexual and physical violence by clients and law enforcement people − Discriminatory laws, criminalization and prosecution of sex work in several countries − Substance abuse People in prison • Prison is a place in which significant sexual violence, such as rape, can occur, which increases the opportunity for STIs to spread • Sexual health services within prisons may be limited People living with HIV • Gonococcal infection increases the likelihood of transmitting and acquiring HIV Indigenous populations • Complex social, cultural and geographic determinants combined with a lack of culturally sensitive health promotion and delivery place Indigenous populations at higher risk of gonococcal infections • In Latin America, high HIV and syphilis prevalence were found in several Indigenous communities • In Australia, the profound consequences of colonisation, racism and intergenerational trauma connected to the Stolen Generation has developed historical barriers and challenges to healthcare access for Aboriginal and Torres Strait Islander communities. In 2016, the gonorrhoea notification rate for Aboriginal and Torres Strait Islander women was 15 times higher than non-Indigenous Australian women. Adolescents and young adults • Overall, gonococcal infections are more common in adolescents and young adults (15–24 years) than in older age groups − Adolescent girls and young women (AGYW) in areas with high HIV prevalence may be disproportionately affected by gonococcal infection and its disease outcomes. For example, gonorrhoea prevalence was estimated at 4.6% among the general population of AGYW in South Africa and 1.7% in Southern/Eastern Africa; it was 8.2% among AGYW at higher risk of infection in East Africa. − Legislations and policies requiring parental consent to access health care, particularly sexual health services, are barriers to access and uptake. − Stigma and discrimination and fear of these also prevent access to health care − Sexual relationships between adolescent girls and older men are shown to contribute to the higher prevalence of STIs in high burden countries − Sensitive and culturally-appropriate sexual history taking should exclude the possibility of sexual abuse Socio-economically disadvantaged populations • In many resource-poor settings, the lack of affordable, feasible tests for diagnosis and screening has led to a higher community prevalence of gonococcal infection • Lower educational opportunities, lack of information, higher unemployment rates, housing conditions or lack of, and other financial hardships limit access to health care and contribute to low prioritization of self-care, including that related to STIs even in the presence of symptoms and signs [7,10,13–30]

Mortality	• Gonococcal infection does not commonly cause death directly, although rare death cases can occur in immunocompromised people with disseminated gonococcal infection − *N. gonorrhoeae* can result in pelvic inflammatory disease (PID) and ectopic pregnancy, which can be life-threatening, particularly in remote or very remote locations • Estimated age-standardised deaths globally was 0.125 per 100,000 (95% uncertainty interval 0.0726–0.156) (2019 data) − Between age 20–24 years, global deaths due to gonococcal infection were estimated at 120 (95% uncertainty interval 93.4–147) per 100,000 for women and 13.1 (8.14–17.4) per 100,000 for men (2019 data) [31,32]

Morbidity	• Most morbidity associated with gonococcal infection is due to ascending genital tract infections in women • If left untreated, sexual health and reproductive consequences include PID, infertility and ectopic pregnancy • However, men are more likely than women to experience symptomatic genital infection, which manifests as urethral discharge syndrome (or less commonly as epididymo-orchitis) Upper genital tract complications • The most common adverse complication of gonococcal infection is upper genital tract infection among women − An estimated 15% of untreated gonococcal infections cause PID, which involves infection of the uterus, fallopian tubes and/or ovaries. Severity varies and can range from subclinical to life-threatening peritonitis − Inflammation and subsequent scarring of fallopian tubes can result in infertility, ectopic pregnancy and chronic pelvic pain ∘ 15–20% of women with gonococcal PID develop infertility ∘ There is a ≥ 50% risk of infertility with ≥ 3 gonococcal infections in women ∘ The risk of upper genital tract scarring is directly proportional to the delay in the treatment of PID ∘ The time it takes to treat gonococcal PID is an important consideration in low-income countries, which may have limited diagnostic and treatment resources and therefore result in higher rates of upper genital tract complications following gonococcal infection Pregnancy complications and mother-to-child transmission • Severe pregnancy complications include preterm birth (odds ratio (OR) 1.55,95% confidence interval (CI) 1.21 to 1.99), spontaneous abortion, intrauterine growth restriction, premature rupture of membranes (OR 1.41, 95% CI 1.02 to 1.92), perinatal mortality (OR 2.16, 95% CI 1.35 to 3.46), and low infant birth weight (OR 1.66, 95% CI 1.12 to 2.48) • Infants born to women with gonorrhoea have a 30–45% increased risk of developing neonatal conjunctivitis, which is a common cause of blindness if left untreated (OR 4.21, 95% CI 1.36 to 13.04) Increased transmission and acquisition of HIV • In both sexes, inflammation due to gonorrhoea might increase the risk for HIV acquisition by two- to three-fold, although there are also important confounding factors to consider at the individual-, sexual network-, service- and societal levels • Co-infection of HIV and gonorrhoea increases HIV viral load which might also increase HIV transmissibility Disseminated gonococcal infection (DGI) • Uncommon • Can manifest as gonococcal bacteraemia, tenosynovitis, septic arthritis, and occasionally gonococcal endocarditis or meningitis [2,7,33–37]

Geographical and seasonal distribution	Geographical distribution • In 2020, WHO estimated that 82 million new cases of gonococcal infection occurred globally • Significant differences in the prevalence of gonococcal infection exist between regions and countries, reflecting variations in sexual practices and networks worldwide. In addition, diagnostic and treatment availability varies between and within countries • Overall, globally, most gonococcal infections occur in LMICs, with the greatest prevalence and incidence rates in low-income countries Seasonal distribution • Evidence suggests gonorrhoea incidence is higher in the warmer months (associated with greater sexual activity in this time period) • For example, an Australian study reported 27% higher odds of urethral gonococcal infection in the first quarter of the year (January-March; summer) for MSM. Data from the USA reported higher cases in autumn and summer for female adolescents [1,17,26,38–43]

Gender distribution	In some settings notification rates of gonococcal infection have increased for both men and women • From 2015–2019, the CDC reported that the gonococcal infection rate for men increased by 60.6% and 43.6% for women (US data). • From 2019–2020, there were greater increases among women (15%) than men (6.6%) reported by the CDC. However, this alteration may be attributed to reduced screening for asymptomatic infections due to COVID-19. Men have a higher incidence rate of gonococcal infection than women globally • In 2020, the global incidence rates of gonococcal infections were 19 cases/1000 women and 23 cases/1000 men • However, the prevalence of infection by gender varies substantially across high, middle and low-income countries • The prevalence of gonococcal infection among women was <0.1% during 2010–2012 in England, 6.6% in South Africa and >14% in antenatal clinics in Papua New Guinea [2,7,17,22,44,45]

Socio-economic status vulnerability(ies) (equity/wealth quintile)	Global estimates demonstrate that the greatest prevalence and incidence rates are in low-income countries or settings • Affordable, feasible tests for gonorrhoea are not available in many LMICs • Those of lower socioeconomic groups have lower educational opportunities and higher unemployment, leading to inaccessibility to resources such as screening, treatment and other relevant health services • Lack of resources could be associated with higher-risk sexual behaviours Strong links between gonococcal infection and those of ethnic minorities, migrants or Indigenous populations with historical barriers to healthcare access • Lack of trust in the healthcare system can exacerbate inaccessibility to sexual health resources [7,13,45,46]

Natural immunity	• There is no naturally acquired protective immunity following *N. gonorrhoeae* infection and re-infection is common • The type of natural immune responses generated after an infection has been a highly debated and controversial area, and the evidence does not provide support for naturally acquired protective immunity • Re-infections are common due to a range of mechanisms, including significant antigenic variation, use of host mimicry and decoy antigens, recruitment of human complement factors and modulation of host immune cells • Reports suggest little variation of anti-gonococcal antibodies in someone previously infected vs no history of infection • There is a concern in the field that exposure to non-protective “decoy” antigens during infection may block subsequent protective immune responses [47–51]

Pathogenic types, strains, and serotypes	• The genomic and antigen diversity of N. gonorrhoeae is very high. Various phenotypic (e.g., serovar determination, antibiotic resistance) and genotypic (e.g., multilocus sequence typing (MLST), *N. gonorrhoeae* multiantigen sequence typing (NG-MAST), whole-genome sequencing) methods have been used to discriminate and type strains of *N. gonorrhoeae.* • In general, there are limited data regarding more pathogenic or virulent strain types, and most strains appear to have the capacity to give similar infections and complications [52].

*Potential indirect impact*	

Antimicrobial resistance (AMR) threat	Since using antimicrobials to treat gonorrhoea, *N. gonorrhoeae* has developed multiple resistance to all antimicrobials that have been recommended against it. • Sulphonamides − By the late 1940s, more than 90% of N. gonorrhoeae isolates had developed resistance • Penicillin − During the mid-1980s, penicillin was officially removed from the first-line anti-gonococcal regimen in the United States due to chromosomally-mediated resistance against penicillin. − Penicillin resistance is now common worldwide. • Tetracycline − In 1985, the first high-level tetracycline-resistant strain was isolated in the US, marking the end of tetracyclines as recommended antibiotics for treating gonorrhoea. − The plasmid that confers the high-level resistance and chromosomally-mediated resistance are now present in strains worldwide. • Spectinomycin − By the mid-1980s, multiple spectinomycin-resistant strains were reported, which led to the discontinuation of spectinomycin as a recommended treatment against gonorrhoea. • Fluoroquinolones − By the mid-to-late-1990s, resistance against high-dose fluoroquinolone had spread across several Western Pacific countries, eventually leading to the removal of fluoroquinolone from being used against gonorrhoea in this region. − The US CDC officially discontinued the recommendation of fluoroquinolone to treat gonococcal infections in 2007. − Today, fluoroquinolone-resistant strains remain common worldwide. • Macrolides − By late-1990s, cases of azithromycin treatment failures had been reported, particularly in South America. − Azithromycin is still recommended in many countries as part of a dual therapy along with ceftriaxone. • Cephalosporins − Currently, two types of cephalosporins are recommended for treatment against gonorrhoea, ceftriaxone (injectable) and cefixime (oral). − Inappropriate use of cephalosporins in Japan from the late-1990s to the early-2000s is hypothesised to have facilitated selection for resistance. − The first extensively drug-resistant (XDR) gonococcal strain was reported in 2011 (H041 from Japan), displaying high-level resistance to ceftriaxone and almost all the antibiotics used against gonorrhoea in the past. − Additional ceftriaxone-resistant strains have since been reported elsewhere but only FC428 and genetically similar subvariants have achieved transmission internationally [53–55].

Epidemic and outbreak potential	• Globally, 82 million new infections of gonorrhoea were estimated among adults in 2020. This is far off the global strategy target regarding progress towards a 90% reduction in gonorrhoea incidence by 2030 (i.e., 8.23 million). • Gonococcal infection prevalence and incidence globally in 2020 are not significantly different from estimated figures in 2016. • However, high-income countries (by World Bank Classification) that have had relatively good control and surveillance of gonorrhoea are now reporting an increasing number of cases: for example, England, European Union/European Economic Area countries, the United States, Australia and some LMICs. This is particularly concerning due to the reported increased gonococcal antimicrobial resistance. • The development and spread of highly resistant strains have the potential to compound the already high burden of disease globally [7,17,22,25,56,57]

Transmission route/potential	• *N. gonorrhoeae* is mainly found in penile discharge, urethra, cervix and vaginal fluid, and can also be found in the oropharynx and anorectum. During sexual intercourse, contact with an infected partner’s penis, vagina, mouth, or anus could lead to the transmission of *N. gonorrhoeae*. • *N. gonorrhoeae* is not specifically carried in semen; ejaculation does not need to occur for infection of gonorrhoea. • Perinatal infection of gonorrhoea can also occur during childbirth when the baby encounters vaginal fluid in the birth canal. As a result, neonates can develop gonococcal conjunctivitis (ophthalmia neonatorum) and blindness if left untreated. Adults may rarely get gonococcal conjunctivitis too [58].

Acquired/herd immunity	• Gonococcal infection does not induce the immune system to develop long-term immunity against it, and repeated infections of *N. gonorrhoeae* are common. • People who have had gonorrhoea before and recovered from the infection can still get infected if they have sexual contact with a person infected with *N. gonorrhoeae* [59].

Co-associated mortality	•STIs like gonorrhoea can enhance the transmission of HIV. − A meta-analysis of 32 longitudinal studies reported a 2- to 3-fold increased risk of HIV infection in patients with gonorrhoea and/or other STIs. − HIV concentration in genital secretions is higher in the presence of STIs. This increase in the availability of viral particles may reflect the accumulation of HIV-infected cells at the site of inflammation (genital mucosa). In HIV-susceptible individuals with gonococcal infection, the availability of HIV-target cells (CD4+ T cells) also increases in the inflamed genital mucosa as part of the immune response against gonorrhoea. • *N. gonorrhoeae* infection causes tubal scarring, tubal occlusion, and the death of ciliated epithelial cells lining the fallopian tube. Due to the damage sustained by the fallopian tube, the risk of ectopic pregnancy and infertility is increased in repeated gonorrhoea infections [60–63]

*Economic burden*	

Health facility costs/out-of-pocket costs/productivity costs	• The estimated total medical cost for gonorrhoea per person in the US in 2016 and 2017 was US$84.95. The mean productivity loss per gonorrhoea case was US$245.70. These cost estimations were adjusted to the value of the 2018 US dollar. In 2018,583,405 cases of gonorrhoea were reported to the CDC. Therefore, medical costs and productivity costs of gonorrhoea combined accounted for about US$193 million. The estimated lifetime medical costs per *N. gonorrhoeae* infection in the United States were $78 ($36-$145) for men and $254 (($96-$518) for women. • There are relatively few estimations for the cost of gonorrhoea in LMICs. Still, it has been suggested that given the higher prevalence in these countries, direct medical costs of gonorrhoea and other STIs will likely be substantial. In an estimation of the costs of implementing the Global STI Strategy in117 LMICs in 2017, the cost of ceftriaxone treatment of symptomatic gonococcal infection alone was US$187 million [64–66].

**Table 2:** Overview of potential target population(s) for gonococcal vaccines and associated delivery strategies.

Target and key populations*	Delivery strategies

Young people (ages 10–24)	• Universal vaccination of young people before first sexual exposure to gonococcal infection would require durable vaccine protection and favourable cost-effectiveness analyses. • Utilization of existing vaccine infrastructure and alignment of the delivery of this vaccine in conjunction with another vaccine, for example: − Gender-neutral, school-based vaccination programs for HPV vaccination − Meningococcal ACWY (MenACWY), diphtheria, tetanus and pertussis (DTaP) and human papillomavirus (HPV) vaccines are provided to school students, and are safe to use with other vaccines; therefore, co-administering with any of these already provided vaccines would be possible • The association of gonococcal vaccines with STIs may affect their acceptability, particularly among parents of adolescents. − Consider legalisation for adolescent self-consent rather than parental consent since the latter might serve as a barrier to access to preventative treatment, particularly in countries with conservative beliefs and attitudes surrounding sex − Consider the provision of counselling and educating parents and families about the risks and long-term effects of gonococcal infection and the benefits of vaccination to facilitate awareness and acceptability of the vaccine − Combine delivery of vaccine with services that provide psychosocial support, developmentally appropriate education, address self-stigma, adolescent peer support groups and networks − Distribution of vaccine information and awareness should be done in a supportive and enabling environment − Utilising internet-/app-based interventions where young people can freely and anonymously express their concerns [7,8,80–83]

Key populations: MSM, transgender people and people living with HIV	• Integrate delivery of vaccine with other STI/HIV prevention programs (including PrEP services); for example, vaccinate MSM presenting for STI screening − In high-income countries, implement various sites of “one-stop-shop”, which includes voluntary STI counselling and testing, STI evaluation and treatment and vaccination − MSM in under-resourced low-income countries may face structural barriers in which they are unable to travel to sexual health services − Consider mobile outreach strategies to increase accessibility • Maintenance of confidentiality is essential, particularly in countries where same-sex practices are less publicly accepted or even criminalised − This might be challenging in rural areas where privacy is more difficult to maintain − Targeted internet-based strategies in which MSM can anonymously access sexual health services and obtain relevant vaccine information might help overcome this barrier • Increase vaccine coverage by increasing vaccine awareness and acceptability • Digital tools − Digital tools have been identified as impactful for improving vaccination rates. Whilst Facebook and YouTube continue to be the most used platforms, Instagram and Tik Tok are especially common among young adults. With such a widespread audience, social media is an excellent low-cost way of disseminating public health messaging related to gonococcal infection and vaccination − Other digital tools, including text message reminders, may be a great cue for action to get vaccinated • Non-digital tools − Distribute information and educate about the risks of gonorrhoea and the benefits of the vaccine at venues where MSM are likely to participate in high-risk behaviours, e.g., bathhouses, baths, tubs, saunas, sex clubs, health clubs, parks, beaches, alleys and public restrooms − Community-level behavioural change includes getting popular leaders of this group to advocate for vaccination to their peers − Individual-level behavioural change includes counsellors meeting one-on-one with at-risk MSM and providing vaccine information • Healthcare workers need to be adequately trained to provide a supportive, non-judgmental and respectful environment whilst providing healthcare for MSM, focusing on addressing and challenging societal discrimination as well as internalised homophobia [84–88]

Key population: Sex workers	• Focusing on providing culturally-competent clinical training for serving sex workers − Educate health providers on the unique needs and vulnerabilities experienced by sex workers • Development of sex-worker specific clinics and facilities where vaccination can take place − Ensure clinic opening times are appropriate for those who work in the sex industry - reports suggest sex workers are unable to visit sexual health clinics during the day as this clashes with their work time • Strategically locating specialised clinics and/or distributing outreach programmes, including vaccination vans in workplaces, such as windows, bars, clubs, saunas, escort services, and private addresses • Maintenance of confidentiality and trust is essential - violation of privacy with their occupation positions sex workers vulnerable to violence and stigmatisation − Evidence suggests sex workers are willing to travel further distances to clinics to avoid being recognised at clinics close to their proximity − This is especially prevalent in areas where sex work is criminalised • Improving vaccine awareness through boosting health literacy via empowerment and education programs - evidence shows the effectiveness of programmes increase when the affected population engage in meaningful participation in their development − Involving community leaders to raise awareness and ensure information is available to hard-to-reach members of the sex work community • Affordability of the vaccine is critical to consider as studies report healthcare workers may charge higher fees once they become aware of their occupation • Shorter intervals between doses of vaccine are associated with better completion of the vaccination schedule [14,89]

Vulnerable population: Incarcerated people	• Integrate delivery of vaccine with other STI/HIV prevention programs, for example, vaccinate people in prison • Due to the rapid turnover of people in prison, an accelerated vaccination schedule is recommended and found to have a clear advantage in the uptake and completion of other vaccines, such as those against hepatitis B virus (HBV). This has been explored in prison settings with a combined hepatitis A virus/HBV vaccination. • Delivery of vaccine should begin promptly after admission to prison • Involve peer educators that participate in training to increase vaccine awareness, promote meaningful participation and empowerment of prisoners [90,91]

Vulnerable populations: Indigenous Populations and other historically disadvantaged populations (ethnic minorities, migrants)	• Self-determination is essential, and strategies surrounding the vaccine implementation should be planned, developed and led by the target communities to meet their economic, social and cultural needs. This includes strong engagement and leadership from community leaders and understanding sensitivities around the use of appropriate language. • Combine the rollout of the gonococcal infection vaccine with the MenACWY, HBV and HPV or vaccination programs or targeted catch-up programs for people aged 20 years and older who missed or were not included in childhood vaccination • Locating services close to where Indigenous people and other historically disadvantaged populations tend to gather could increase attendance and engagement with health programs [92]

* Key populations are disproportionately affected in most contexts. Vulnerable populations are at higher risk for gonococcal infection in certain situations or contexts that may vary between and within countries.

**Table 3:** Overview of non-commercial stakeholders engaged, their interest and potential demand for gonococcal vaccines.

Stakeholders engaged	Summary of position/interest	Potential demand and uptake	References

WHO	The rapidly changing antimicrobial susceptibility of *N. gonorrhoeae* has created an important public health problem.	*N. gonorrhoeae* is on WHO’s priority list of AMR pathogens, categorised as a high-priority pathogen for research and development efforts.	WHO Global Health Sector Strategies on HIV, Virus Hepatitis and STIs, 2022–2030
	The WHO Global Health Sector Strategies on HIV, Viral Hepatitis and STIs, 2022–2030 recognises *N. gonorrhoeae* as one of the main STIs requiring immediate action for control due to the rising risk of untreatable gonorrhoea and risk of coinfection with other STIs.	The WHO Global Health Sector Strategies on HIV, Viral Hepatitis and STIs outlines the need to accelerate access to innovations by developing new preventive interventions, including vaccines. The WHO PPCs for gonococcal vaccines provides guidance on WHO’s preferences for emerging gonococcal vaccines, including from the perspective of low to middle-income countries.	WHO Global Action Plan to Control the Spread and Impact of AMR in *Neisseria gonorrhoeae*, 2012 Global Health Sector Strategy on WHO Global Gonococcal Antimicrobial Surveillance Programme (GASP) [3,7,93–95]

Wellcome Trust (supported by Boston Consulting Group)	The case for developing a vaccine targeting *N. gonorrhoeae* is strong due to high incidence, high morbidity, and current circulation of resistant strains. Although significant development challenges remain, evidence of MenB vaccine cross-protection has fostered fresh optimism in the expert community.	AMR is a major concern globally, with the treatment of *N. gonorrhoeae* becoming increasingly challenging. Vaccines play an important role in reducing the demand for antibiotics, mitigating the risks for the inadequacy of the current pipeline.	Boston Consulting Group-Wellcome Report on Vaccines to Tackle Drug Resistant Infections [97]

Pan American Health Organization (PAHO)	*Neisseria gonorrhoeae* Antimicrobial Resistance Surveillance: Consolidated Guidance Prevention, early diagnosis, and effective treatment are essential for the control and elimination of *N. gonorrhoeae* as a public health problem. An epidemiological alert was issued by PAHO in 2018 regarding the emerging cephalosporin resistance in *N. gonorrhoeae*, advising countries to strengthen AMR prevention.	PAHO serves as the Regional Office for the Americas of the WHO and has worked alongside WHO to develop the Global Health Sector Strategies on HIV, viral hepatitis and STIs and the WHO preferred product characteristics for gonococcal vaccines.	[96]

European Commission European Centre for Disease Prevention and Control (ECDC) The European Gonorrhoea Response Plan Group	European Gonococcal Antimicrobial Surveillance Programme has reported gonococcal infections with resistance to cefixime and ceftriaxone, particularly a rapidly increasing resistance to azithromycin. The spread of resistance against cephalosporins, the main recommended antibiotic class for gonorrhoea therapy across Europe, is extremely concerning.	In the absence of an effective vaccine, condom use, early diagnosis and treatment, and other conventional public health measures remain the main public health strategies to interrupt transmission and avoid co-morbidities of gonococcal infection. The response plan for multi-drug resistant and XDR gonorrhoea developed by the ECDC focuses on strengthening AMR surveillance, treatment failure monitoring and establishing a communication strategy to inform European authorities of the results from AMR surveillance.	[98,99]

U.S. Federal Task Force on Combating Antibiotic-Resistant Bacteria	The National Strategy for Combating Antibiotic Resistant Bacteria identifies priorities and coordinates investments to prevent, detect, and control outbreaks of resistant pathogens recognized by CDC as urgent or serious threats, including ceftriaxone-resistant *N. gonorrhoeae*. Effective vaccines may potentially reduce the need for antibiotics and prevent resistance from developing in the first place.	The National Action Plan for Combating Antibiotic-Resistant Bacteria, 2020–2025 states the US government will pursue activities to enhance translational and clinical research on vaccines for antibiotic-resistant bacteria	[100,101]

National Institute of Allergy and Infectious Diseases (NIAID), U.S.	The National Institute for Allergy and Infectious Diseases (NIAID) has continuously been engaging with WHO to address the gaps in knowledge about advancing STI vaccine development, including vaccines for *N. gonorrhoeae*. In 2015, NIAID sponsored the workshop “Gonorrhea Vaccines: the Way Forward” to discuss key questions on the current status of gonorrhoea vaccine research and the path forward. The workshop identified that broader access to in vitro assays, reagents, and animal models could potentially improve collaboration and acceleration of the vaccine development process. Efforts to refine existing animal models and leverage the experimental human male infection model for phase I/II trials should also continue. In addition, continuing clinical studies can help develop a better understanding of the natural history of gonococcal infection.	The National Institute for Allergy and Infectious Diseases has been working with WHO to implement the Global STI Vaccine Roadmap, [106] which includes key activities to advance gonococcal vaccine development. One such activity is developing WHO PPCs to reflect WHO guidance on desired vaccine parameters to meet priority public health goals, especially for LMICs.	US NIAID Workshop: Gonorrhoea Vaccines: the Way Forward, 2015 [102]

National Institute for Communicable Diseases/National Health Laboratory Services STI Reference Centre	The NICD/NHLS plays a leading role in sub-Saharan Africa’s WHO Gonococcal Antimicrobial Surveillance Programme.	Within South Africa, the STI Reference Centre at NICD/NHLS developed and has coordinated the National Microbiological Surveillance Programme for STIs since 2005. Local AMR gonococcal surveillance across the nine provinces of South Africa (since 2006) has informed STI management and control guidelines. Surveys have confirmed widespread ciprofloxacin resistance among gonococci. Renewed efforts from WHO and NICD/NHLS to develop African GASP started in 2010. The STI Reference Centre designed and implemented a protocol to determine gonococcal resistance in gonococci isolated from men with urethral discharge in Zimbabwe. Site visits were also undertaken to the National Reference Laboratory in Madagascar and Tanzania to discuss survey protocol issues and training needs.	[103]

Australian Government Department of Health	The Australian Government Department of Health provides leadership for coordinating the national response to blood-borne viruses and STIs through a number of national strategies and initiatives. New technologies and innovative approaches such as vaccines are required to enhance the impact and produce better health outcomes for people with or who are at risk of STIs.	The National Antimicrobial Resistance Strategy 2020 states that an effective research response to antimicrobial resistance needs to include research of therapeutic alternatives to antimicrobials, such as vaccines. Further research is the only way to inform the development of improved preventative measures.	[104,105]

**Table 4:** Summary of existing guidance on preferences for product attributes of gonococcal vaccines intended for use in LMICs.

Parameter	Preferred characteristic	Notes for gonococcal vaccines	Additional notes for MenB vaccines with potential cross-protection^1^

Vaccine type	Gonococcus-specific vaccine.	Vaccines specifically formulated to optimize efficacy against gonococcal infection and related adverse SRH outcomes^2^ are preferred. Although several potential candidate gonococcal antigens exist, as of 2020 no vaccines designed *de novo* for gonococcal infection were in clinical trials. As a result, the product development pathway for gonococcus-specific vaccines is still long, possibly 10–12 years. Gonococcal vaccines must be suitable for use globally because substantial numbers of gonococcal infections occur in all countries, regardless of their stage of economic development.	In observational studies, OMV-based MenB vaccines appeared to provide cross-protection against *Neisseria gonorrhoeae* with an estimated vaccine effectiveness of 20–30% in preventing gonococcal infection and related hospitalizations. A MenB vaccine with an indication to prevent gonococcal infection and/or disease may be available well before a licensed gonococcus-specific vaccine, and therefore may provide an earlier intervention for gonococcal prevention and control. In addition to evaluating existing licensed OMV-based MenB vaccines in ongoing trials, other options include developing new meningococcal vaccines that provide greater cross-protection against gonococcal infection and disease.

Vaccine indication	Prevention of gonococcal infection.	The ultimate, long-term goals of gonococcal vaccines are to prevent adverse SRH outcomes and reduce the impact of gonococcal AMR. These goals will best be accomplished by the indication of preventing gonococcal infection, for the following reasons: • Most gonococcal infections are asymptomatic but can still lead to adverse SRH outcomes, particularly in women; and • A vaccine may show efficacy in a trial with a disease endpoint but leave residual asymptomatic infections that could still lead to adverse SRH outcomes or propagate AMR. Existing molecular diagnostic assays can easily and accurately measure gonococcal infection as an outcome variable in Phase III clinical trials, including at different sites of infection. Many gonococcal disease outcomes, such as uppergenital tract infections and complications in women, are more difficult to measure. Collecting data on measurable disease outcomes in clinical trials, such as symptomatic gonococcal urethritis in men, may offer additional indications beyond infection. Vaccine impact on most adverse SRH outcomes and AMR would likely be difficult to demonstrate in pre-licensure clinical testing. Consideration should be given to collecting supporting evidence for a positive impact on these outcomes during pre-licensure studies and designing post-licensure studies to evaluate them.	Planned clinical trials to determine the specific efficacy of OMV-based MenB vaccines to prevent gonococcal infection can provide insight on clinical endpoints for measuring gonococcal infection and other short-term disease outcomes; for example, the relative incidence of different outcomes according to anatomical site (urogenital, oropharyngeal and/or rectal). If already licensed OMV-based MenB vaccines show some efficacy against gonococcal infection, measuring their impact on adverse SRH outcomes and AMR might be done sooner and in parallel with developing more optimized gonococcal vaccines.

Target populations	Young people AND/OR Specific populations at higher risk for gonococcal infection.	WHO defines young people as those between the ages of 10 and 24 years, including adolescents (10–19 years) and young adults (20–24 years). Specific populations at higher risk for gonococcal infection are defined here in two categories: key and vulnerable populations. • Key populations for gonococcal infection are disproportionately affected in most contexts and include men who have sex with men (MSM), sex workers, transgender people and people living with HIV (PLHIV). • Vulnerable populations are at higher risk for gonococcal infection in certain situations or contexts that may vary between and within countries. Some examples include: incarcerated people; ethnic minorities or Indigenous populations with historical barriers to healthcare access; and migrants or young people living in communities with known high rates of gonococcal infection or HIV, especially young women, who have the greatest risk of adverse SRH consequences from gonococcal infection. The choice of broad-based vaccination of young people and/or targeted vaccination of specific populations at higher risk for gonococcal infection in different settings will depend on factors such as: • gonococcal epidemiology • vaccine efficacy in those with prior infection • duration of vaccine protection • cost-effectiveness analyses • programmatic considerations (see “Vaccine delivery strategy” below). These factors can also help to refine the precise age range to be targeted among young people. Universal vaccination of young people before first sexual exposure to gonococcal infection, and aligned with existing vaccine delivery infrastructure, would be ideal, but would require durable vaccine protection and favourable cost-effectiveness analyses. It is currently unknown whether prior gonococcal infection will affect gonococcal vaccine efficacy, which may influence vaccine effectiveness among higher-risk populations. Gender-neutral vaccination is desirable, because gonococcal infections can lead to disease consequences in all people, infections in all people contribute to, and are affected by, AMR, and for general equity reasons.	The incidence of invasive MenB disease has substantial variability by geographic location and over time. The highest incidence is among infants, but disease occurs at all ages. Outbreaks can affect multiple age groups. In HICs that use MenB vaccines, infant vaccination is emphasized; however, a few countries recommend MenB vaccines for young people living in areas where close contact is frequent (for example, people entering university or the military) or for special populations. These populations may overlap with potential target populations for gonococcal vaccines. Many LMICs with a high prevalence of gonococcal infection in the general population either do not appear to have significant MenB disease incidence or do not utilize MenB vaccine widely because the cost is prohibitive. Better data are needed to understand the epidemiological overlap and populations at risk for both pathogens, as well as to understand the factors contributing to MenB vaccine use in different settings. If the preferred target populations in a setting comprise only a small proportion of the population, expanding an existing licensed vaccine may be more favourable economically than developing a *de novo* vaccine for that group.

Vaccine efficacy	50 to 70% efficacy or greater.	Existing mathematical models suggest that a vaccine with 50% efficacy or greater could have a marked effect in reducing gonococcal infection prevalence at a population level, particularly if vaccine coverage is high and protection lasts through periods of highest gonococcal infection risk. Preferred vaccine efficacy levels will be refined with updated vaccine impact models, input from key stakeholders, and further information about likely vaccine characteristics from ongoing research. If MenB vaccines are found to provide cross-protection and are being used for prevention of gonococcal infection, gonococcus-specific vaccines should have significantly superior efficacy.	Observational studies suggest the effectiveness of an OMV-based MenB vaccine against gonococcal infections may be 20–30%, which models predict could still have a substantial effect in reducing prevalence of gonococcal infection in the population. A lower efficacy could be acceptable for broadening use of MenB vaccines to prevent gonococcal infection compared with use of a standalone gonococcal vaccine, given an existing indication for MenB disease prevention, particularly in countries with a history of MenB endemic disease and/or outbreaks.

Duration of protection	The long-term goal is at least 10–15 years’ duration for vaccinating young adolescents without a booster. However, shorter durations of protection (e.g. 3–5 years) could still provide benefits for older age groups and specific populations at higher risk.	Ideally, vaccine-induced protection from gonococcal infection should last throughout the timeframe of highest risk of infection. Therefore, the optimal duration of protection depends on target age. In most settings, peak incidence appears to be in young adults (20–24 years). For young adolescents (10–14 years), duration of protection might need to be 10–15 years or more to cover the period of peak incidence, or a booster dose may be needed. However, for older adolescents or young adults, shorter durations of protection could cover the period of highest risk. Periods of high risk may be observed over shorter time frames, such as during the use of PrEP for HIV prevention. Therefore, for some populations at higher risk, a duration of protection of only 3–5 years may still have substantial benefits. Duration of vaccine protection will likely not be known at the time of initial licensure. Although the aspirational, long-term goal for duration of protection is at least 10–15 years, first-generation vaccines may have shorter durations of protection: for example, 3–5 years. Modelling of vaccine impact and cost-effectiveness can guide use of initial vaccines with potentially limited duration of protection in different epidemiologic settings. Research to determine correlates of protection will be valuable for predicting and assessing duration of protection, as will post-licensure effectiveness data.	The duration of protection of existing MenB vaccines against MenB disease is unknown, but most likely around 36 months following three doses in infancy. Similarly short-lived protection has been suggested in observational studies of MenB vaccines to prevent gonococcal infection. Therefore, one or more booster doses may be required to cover the period of highest risk for gonococcal infection.

Vaccine delivery strategy	Young people: alignment with existing vaccine delivery infrastructure. Populations at higher risk: integration with HIV prevention programmes and other SRH services.	The most appropriate vaccine delivery strategies in different settings will be determined by target populations, vaccine characteristics, and related health systems and programmatic factors. Universal vaccination of young people may be more straightforward programmatically than delivering vaccine in a targeted fashion to higher-risk populations, who may be harder to identify and reach. Gonococcal vaccination in early adolescence would allow use of existing adolescent vaccine delivery infrastructure. However, the effectiveness of this approach would depend on duration of vaccine protection. In settings where gonococcal infection incidenceis low in the general population but concentrated within specific populations at higher risk, a more focused vaccination programme might more efficiently interrupt community-wide transmission, depending on how easily these populations can be reached. Demand sizing and evaluation of care-seeking patterns of specific target populations will be informative. Although targeted vaccination programmes have been difficult to implement in the past, the expansion of HIV prevention programmes and other SRH services, such as increasing use of PrEP and outreach programmes for key populations, might offer novel opportunities to deliver more focused gonococcal vaccination. Communication, community outreach and marketing strategies regarding gonococcal vaccines should be considered in advance. Unlike HPV vaccines, which are widely seen as cancer-prevention vaccines, gonococcal vaccines will likely be more clearly associated with a sexually transmitted infection, which may affect acceptability, particularly to parents of adolescents.	In settings where target populations for MenB vaccine already include young people, such as those entering university or military recruits, vaccinating with an OMV-based MenB vaccine to also prevent gonococcal infection would be relatively straightforward. Adoption and uptake of existing MenB vaccines remain relatively low globally. Expanding the indication of an existing MenB vaccine to include gonococcal infection prevention could make this vaccine more cost-effective and may affect the decision to introduce MenB vaccines in more countries and in new target populations. Meningitis may be perceived as less stigmatizing than gonococcal infection. Initial promotion of the use of MenB vaccines with some potential to prevent gonococcal infection among adolescents may increase acceptability of a specific gonococcal vaccine later.

Route of vaccine administration	Oral or parenteral delivery.	Local mucosal immunity likely plays an important role in protection against gonococcal infection. An oral mucosal route is preferred for ease of administration in LMIC settings. Mucosal delivery via other routes, such as intranasal, might induce appropriate immune responses but will be more difficult to deploy, particularly in resource-constrained settings. Parenteraly routes of administration include intramuscular and subcutaneous injections and intradermal routes, which can be needle-free, such as via a transdermal or microarray patch. Needle-free methods are preferred for ease of administration. Vaccine presentation and stability characteristics that facilitate storage and deployment in LMIC settings should also be considered.	Observational studies of OMV-based MenB vaccines suggest that protective mucosal immunity against gonococcal infection can result from a parenteral vaccine.

Adjuvant	Preference for no adjuvant unless required for immunogenicity.	Adjuvant could be included if proven enhancement of vaccine immunogenicity and efficacy is demonstrated in primary target populations. Adjuvant formulations with previously demonstrated safety profiles in the target population are likely to be well tolerated.	Existing licensed OMV-based MenB vaccines are adjuvanted with aluminium hydroxide.

Schedule	Ideally, up to two doses for primary immunization.	Depending on the vaccine platform and formulation, two to three doses may be required for strong and durable immunity. Research should determine the requirements for alternative primary dosing or booster doses. This might be post-licensure, as for HPV vaccines. If more than one dose is needed, aligning the dosing schedule with existing delivery platforms, such as the vaccine delivery schedule for HPV or meningococcal vaccination, is preferable.	The typical vaccination schedule for available OMV-based MenB vaccines is a two-dose primary series, followed by a booster dose at 1 year of age for infants and a two-dose primary series for older ages, including adolescents and young adults. Because the period of highest risk for gonococcal infection is typically longer than the risk period for meningococcal infection, a booster dose will likely be needed ifthis vaccine is also intended to prevent gonococcal infection over longer periods.

Safety	A safety and reactogenicity profile at least as favourable as other WHO-recommended routine vaccines.	A favourable safety profile will need to be demonstrated in adults before progressing to vaccination of young adolescents, as was done for HPV vaccine.	The safety profile of licensed MenB vaccines has been established.

Concomitant use	Demonstration of favourable safety and immunologic noninterference upon coadministration with other vaccines recommended for use.	Lack of clinically important interference in immunogenicity for gonococcal vaccines and for co-administered vaccines, as well as the safety of co-administration, should be documented in post-licensure studies. Evidence should be collected on the ability to coadminister gonococcal vaccines with other vaccines given in similar target populations. for example, HPV; tetanus, diphtheria and acellular pertussis (Tdap); and meningitis vaccines in adolescents and young adults.	If OMV-based MenB vaccines are found to show cross-protection against gonococcal infection, evidence should be collected to evaluate for clinically important interference with this effect when MenB vaccines are co-administered with other vaccines.

Value assessment and affordability	The vaccine should be cost-effective and price should not be a barrier to access, including in LMICs. Dosage, regimen and cost of goods should be amenable to affordable supply.	Obtaining more comprehensive data on the burden of adverse health outcomes related to gonococcal infection, including in LMICs, will allow more precise quantification of the full potential value of gonococcal vaccines. The value of gonococcal vaccines is heavily influenced by increasing gonococcal AMR and its predicted effect on clinical treatment failures, the costs of treatment, and increases in gonococcal infection and disease outcomes.	The cost-effectiveness of existing MenB vaccines would become more favourable if they are found to prevent gonococcal infection in addition to MenB disease.

Prequalification and programmatic suitability	The vaccine should be prequalified according to the WHO process outlined [107].	WHO-defined criteria for programmatic suitability of vaccines should be met [107].	

1 Many of the notes in the “gonococcal vaccine” column also apply to a MenB vaccine with an added indication to prevent gonococcal infection. This column is intended to supplement the notes with additional considerations, or how the preferred characteristics might be thought about differently if a licensed MenB vaccine shows some efficacy against gonococcal infection and/or disease.

2 Adverse SRH outcomes related to gonococcal infection include acute gonococcal disease (e.g. urethritis, cervicitis, proctitis, pharyngitis, conjunctivitis) AND complications of initial infection affecting SRH (e.g. pelvic inflammatory disease, infertility, ectopic pregnancy, chronic pelvic pain, epididymo-orchitis) AND associated adverse clinical outcomes of pregnancy (e.g. chorioamnionitis, premature rupture of membranes, preterm birth, neonatal conjunctivitis) AND gonococcal-related HIV acquisition and transmission AND rarer complications (e.g. disseminated gonococcal infection, urethral stricture).

**Table 5:** Overview of parameters that inform the scientific feasibility of developing an effective gonococcal vaccine for LMIC public market use.

Parameter	Issues and evidence

Diagnosis/case ascertainment	• NAATs are highly sensitive and specific and the gold standard for diagnosing *N. gonorrhoeae.* However, NAATs are inaccessible in many LMICs due to cost, time to obtain results, and the need for resources, training and infrastructure. • In most of the world, particularly LMICs, *N. gonorrhoeae* diagnosis relies on a syndromic approach. This is considered relatively adequate for urethral discharge, but syndromic management has low diagnostic accuracy for vaginal, anal and pharyngeal infections due to the high level of asymptomatic infections at these sites. • Gram stain microscopy is inexpensive and reliable for diagnosing *N. gonorrhoeae* urethritis in men, but it is not available in all settings and is not sufficiently reliable for infection in women or extragenital infection [7,8,110].

Biomarkers/Correlates of risk and/or protection	• There is no established correlate or biomarker. • There is no protective immunity to natural infection, and repeat infections are common • There are no known immunologic surrogates or correlates of protection • Enzyme-linked immunosorbent assay (ELISA), serum bactericidal activity (SBA), opsonophagocytic killing (OPK) and target function inhibition assays have been developed and are used for the evaluation of immunogenicity of *N. gonorrhoeae* vaccine candidates in animals and to evaluate the functional activity of vaccine-induced antibodies. These assays may be relevant once a correlate of protection is identified. SBA is the correlate of protection for the closely related bacteria *N. meningitides* [7,8,49,51,111–113].

Sero-epidemiological data	Ongoing clinical trials with 4CMenB, and related research, have found that: • Humans develop humoral and cellular immune responses following infections; however, in most cases, these do not protect from reinfection and have not led to the identification of a correlate of protection. • *N. meningitidis* serogroup B OMV-based vaccine may induce cross-reactive protection against *N. gonorrhoeae*, and clinical trials are underway to determine the efficacy of the 4CMenB vaccine against *N. gonorrhoeae*. Some of these trials are evaluating the immune response to *N. gonorrhoeae* following vaccination to identify a correlate of protection [4,49,114].

Clinical endpoints	• Infection is likely to be the endpoint as there are complications with measuring disease outcomes (time to presentation, imprecision in diagnosing disease, the similarity between disease caused by other STIs such as *Chlamydia trachomatis*). • The WHO PPC for gonococcal vaccines suggests that “possible indications for gonococcal vaccines include prevention of gonococcal infection (both asymptomatic and symptomatic) and/or specific gonococcal-associated disease outcomes, such as symptomatic urethritis, cervicitis or PID. It is presumed that vaccines preventing gonococcal infection will ultimately prevent the adverse sexual and reproductive health consequences and AMR associated with those infections.” • The reduction in gonococcal infections as measured by NAATs may be the most feasible primary clinical endpoint for clinical trials. • The use of a disease outcome (e.g., symptomatic urethritis, cervicitis, PID or infertility) is associated with several potential complications. Many *N. gonorrhoeae* infections are asymptomatic but may still lead to adverse outcomes, particularly in women, which are often difficult to measure and confidently ascribe an aetiology to (e.g., PID) and may take years to develop or become apparent following infection (e.g., infertility). Asymptomatic infections may also still propagate AMR [7,8].

Controlled human infection model (CHIM)	• A controlled human infection model (CHIM) of male urethral gonorrhoea has been used to investigate *N. gonorrhoeae* pathogenesis and immune responses to *N. gonorrhoeae* in >200 participants. • The CHIM replicates the early stages of natural urethral infection in men (approximately 1–6 days) before infection needs to be treated. • The CHIM is not widely available, is expensive and complicated to scale up, and is only used for male urethral infection due to the potential severe sequelae resulting from female genital tract infection. However, it has been suggested that a CHIM could be developed for other participants (e.g., postmenopausal women) [7,8,115–117].

Opportunity for innovative clinical trial designs	• The WHO PPC for gonococcal vaccines suggests that “given the lack of currently known immune correlates of protection, a large clinical end-point trial will, in all likelihood, be required for gonococcal vaccines” • Current clinical trials of the licensed meningococcal group B vaccine 4CMenB for preventing *N. gonorrhoeae* infections may be indicative of future efficacy trials (see Table 6; NB. 4CMenB has a predicted efficacy of ~30% against *N. gonorrhoeae*). • One study aims to recruit 2200 individuals, both men and women 18–50 years old, who are particularly vulnerable to gonococcal infection • Another study aims to recruit 730 participants who are gay and/or bisexual men, either HIV-negative and taking PrEP or HIV-positive with undetectable viral load, who are at high risk of gonococcal infection [7,108,120].

Regulatory approach(es), including potential accelerated approval strategies	• Global estimates indicate that most gonococcal infections occur in LMICs. However, epidemiology varies by geographical location, age and risk group and high prevalence and incidence are seen in key and vulnerable populations in HIC. Therefore, ideally a vaccine would be licensed in both LMICs and HICs. • The vaccine should be pre-qualified according to the WHO process outlined. • Licensure will likely be based on efficacy data in Phase 3 clinical trials • CHIMs may be useful in demonstrating protection [7,8,45,118].

Potential for combination with other vaccines	• Combination of a *N. gonorrhoeae* vaccine with other vaccines would be possible and may depend on the target population considered • The WHO PPCs for gonococcal vaccines lists young people and/or specific populations at higher risk for gonococcal infection as key target groups. − For young people - combination with vaccines in the routine immunisation schedule could be feasible (e.g., HPV; tetanus, diphtheria and acellular pertussis (Tdap); and meningococcal vaccines in adolescents and young adults) − For populations at higher risk, the combination with HPV and HBV vaccines (or other STI vaccines once available) could be feasible [7]

Feasibility of meeting presentation and stability requirements	• Vaccine presentation and stability characteristics that facilitate storage and deployment in LMIC settings should be considered. • There are no apparent barriers to meeting feasibility requirements based on the types of vaccine candidates and vaccine platforms in preclinical and clinical development (see Table 6) [7].

Vaccine platform	• Likely to be a recombinant protein, several recombination proteins or OMV; based on preclinical work so far, all should be feasible • There are no apparent barriers to large-scale manufacturing based on the types of vaccine candidates and vaccine platforms in preclinical and clinical development (see Table 6).

Large scale manufacturer capacity / interest	• Interest in gonococcal vaccine development has been reinvigorated not only by the marked increases in gonococcal AMR but also by mounting scientific data suggesting gonococcal vaccines are biologically feasible, particularly observational studies suggesting that OMV-based *N. meningitidis* serogroup B (MenB) vaccines may provide cross-protection against *N. gonorrhoeae* [7].

**Table 6:** Overview of candidate vaccines for gonococcal infection in clinical trials.

Candidate	Antigen platform	Developer/manufacturer	Phase of development, population, and location	Route of administration, no. of doses, schedule	Presentation and stability	Clinical trial references
**4CMenB (Bexsero)**	*Neisseria* *meningitidis* group B outer membrane vesicle (OMV) and recombinant protein vaccine	GSK	• Phase 4 • 15 participants aged 18–25 years, non-pregnant, HlV negative, no history of immunologic disorder, not on immunosuppressive drugs • North Carolina, USA	• Deltoid intramuscular (IM) injection • Two doses of 0.5 ml • Day 0 and week 5	• Prefilled glass syringe with 0.5ml suspension; one dose per carton or ten doses per carton • Store at 2–8 °C, freezing prohibited, protection from light required.	[117]
**4CMenB (Bexsero)**	Same as above	GSK	• Phase 4: prospective cohort + case-control study of vaccine effectiveness against gonorrhoea • 5,000 persons aged 14–19 years • Northern Territory, Australia	• Deltoid lM injection • Two doses of 0.5 ml • Day 0 and month 2	• Same as above	[119]
**4CMenB (Bexsero)**	Same as above	GSK	• Phase 3 randomized controlled trial (RCT) • 730 men who have sex with men (MSM), taking HlV pre-exposure prophylaxis (PrEP) or living with HlV with undetectable viral load • Public sexual health clinics in NSW, VIC, and QLD, Australia	• Deltoid lM injection • Two doses of 0.5 ml • Day 0 and month 3	• Same as above	[120]
**4CMenB (Bexsero)**	Same as above	GSK	• Phase 3 RCT • 502 MSM over 18 years old and taking HlV PrEP • Hospitals in Paris, France	• Deltoid lM injection • Two doses of 0.5 ml • Day 0 and month 2	• Same as above	[121]
**4CMenB (Bexsero)**	Same as above	GSK	• Phase 3 • 112 MSM aged 18–50 years • Gold Coast University Hospital, Queensland, Australia	• Deltoid lM injection • Two doses of 0.5 ml • Day 0 and month 3	• Same as above	[122]
**4CMenB (Bexsero)**	Same as above	GSK	• Phase 2 RCT • 2200 men and women aged 18–50 years, who are particularly vulnerable to gonococcal infection • Alabama, Georgia, Louisiana, Maryland, USA, and Thailand	• Deltoid lM injection • Two doses of 0.5 ml • Day 0 and month 2	• Same as above	[108]
**4CMenB (Bexsero)**	Same as above	GSK	• Phase 2 mechanistic clinical trial • 50 participants, male and female aged 18–49 years • Georgia, USA	• Deltoid lM injection • Two doses of 0.5 ml • Day 1 and day 29	• Same as above	[123]
**NgG**	*Neisseria gonorrhoeae* generalized modules for membrane antigens (GMMA) vaccine	GSK	• Phase 1/2 • 774 men and women 18–50 years old at risk of gonococcal infection • 8 countries: USA, UK, France, Germany, Spain, Brazil, Philippines, South Africa	• IM injection • Phase 1: 2 doses of low, medium, or high dose • Phase 2: 2 doses of highest tolerated dose from Phase 1	• Details not available	[124]

**Table 7:** Overview of population-based and modelling studies on gonococcal vaccines that measure health impact on disease burden and transmission.

Policy question	Assessment method/measure	Additional information specific to models	Assumptions	Outcomes/interpretation
*Population-based studies*				
What is the effectiveness of the outer membrane vesicle meningococcal B vaccine (MeNZB) against gonorrhoea? [4]	Vaccine effectiveness was measured through laboratory isolation or detection of *N. gonorrhoeae* The odds ratio (OR) was estimated by comparing disease outcomes in vaccinated individuals versus unvaccinated individuals via multivariable logistic regression. Vaccine effectiveness was calculated as 100×(1–oR).	For the primary analysis, cases were those who were gonorrhoea-positive only, and controls were chlamydia-positive only Those with a co-infection of both gonorrhoea and chlamydia could be assigned to either case or control group, and so a sensitivity analysis was conducted to identify how much inclusion of these individuals would impact the estimate of MeNZB effectiveness Multivariate logistic regression including age group, ethnicity, sex, geographical location and deprivation quintile was used to provide an adjusted estimate of vaccine effectiveness	Target population included young adults aged 15–30 years old based in New Zealand Complete vaccination required three doses of vaccine at least 6 months before laboratory confirmation of gonorrhoea MeNZB programme achieved social and ethnic equity in vaccine coverage	Estimated vaccine effectiveness of MeNZB against gonorrhoea after adjustment for ethnicity, deprivation, geographical area, and sex was 31% (95% CI 21–39) Estimated vaccine effectiveness of MeNZB against hospitalization caused by gonorrhoea after adjustment for gender, ethnicity, and deprivation was estimated to be 24% (95% CI 1–42%) Co-infection with chlamydia was associated with lower vaccine effectiveness The potential ability of the MeNZB vaccine to provide even modest protection against gonorrhoea would have substantial public health benefits, given the prevalence of gonorrhoea
What is the effectiveness of a 4CMenB vaccine against gonorrhoea? [5]	Identified and linked confirmed gonorrhoea case records with immunization registry records Effectiveness of the vaccine was calculated by using infection-level data to compare the prevalence of *N. gonorrhoeae* infection during vaccinated and unvaccinated periods This analysis was conducted via a multivariable analysis to determine unadjusted and adjusted prevalence ratios (APR) and 95% CI using log-binomial regression with generalised estimating equations to account for correlations between multiple STI infections overtime per person Vaccine effectiveness was calculated as 100x (1-APR)	Sensitivity analysis was conducted to assess vaccine effectiveness assuming a 6 or 12-month duration of protection Variables that were adjusted for in the model included race/ethnicity, gender and jurisdiction Secondary analysis was conducted to estimate vaccine effectiveness for gonorrhoea/chlamydia co-infections by using a multivariable analysis to calculate adjusted prevalence ratios	Target population was young adults aged 16–23 years old in New York and Philadelphia Complete vaccination required two doses, 30–180 days apart Post-vaccination immunity started 30 days after receipt of the complete or partial vaccination series Vaccine protection duration was assumed to be 12 months	Complete vaccination was 40% (95%CI: 23–53) effective against gonorrhoea This study showed that the 4CMenB might offer cross-protection against *N. gonorrhoeae* and provide further evidence supporting the feasibility of an effective gonococcal vaccine.
What is the vaccine effectiveness and impact on MenB and gonorrhoea at two years? [6,125]	Vaccine effectiveness was estimated by the reduction in the odds of infection using the screening and case-control methods Vaccine impact was estimated by comparing disease incidence pre-and-post 4CMenB program in eligible vaccine cohorts using Poisson or negative binomial models	All gonorrhoea-positive cases included those who had or did not have chlamydia co-infection. Because co-infection may alter data, a sensitivity analysis was conducted to estimate gonorrhoea only infection (excluding co-infected gonorrhoea cases) Vaccine impact may have been confounded by public health strategies implemented during the COVID-19 pandemic	Target population was adolescents and young adults in South Australia Complete vaccination included two doses Vaccine coverage data was obtained from the Australian Immunisation Register	This study supports evidence that the MenB can provide cross-protection against gonorrhoea in adolescents and young adults over three years of observation. Vaccine impact 30% reduction in the incidence of gonorrhoea in adolescents aged 15–17 years (IRR: 0.70, 95%CI: 0.43–1.14, p=0.152) Vaccine effectiveness VE = 37% (OR: 0.63, 95%CI: 0.50–0.80, p<0.001) for two doses
*Modelling studies*				
What aspects (e.g., vaccine efficacy, duration of protection, and coverage levels) of potential gonococcal vaccines have an impact on disease prevalence? [82]	Model adapted from an individual-based model developed for a chlamydia vaccine Simulated a vaccination program in which vaccination takes place at 13 years of age	Gonorrhoea-specific parameters were incorporated into the model Calibration meant the mean prevalence of gonococcal infection in the absence of a vaccine was 1.6–1.7% Per-exposure probability of a woman infecting a man was 0.28, and the probability for a man infecting a woman was 0.50	Target population included a sexually active heterosexual population and allowed for both ongoing regular and casual (short-term) partnerships This model considered vaccines with efficacies of 10–100% and durations of 2.5–20 years Assumed no immunity after the resolution of an infection 100% vaccine coverage	Model simulations predicted a 90% reduction in gonococcal infection prevalence after 20 years if all 13-year-olds were given a non-waning vaccine with 50% efficacy or a vaccine with 100% efficacy that wanes after 7.5 years A 40% reduction in prevalence is achievable with a non-waning vaccine of only 20% efficacy A vaccine of moderate efficacy and duration could have a substantive impact on the gonococcal prevalence and disease sequelae if coverage is high and the protection lasts over the highest risk period (i.e., most sexual partner change) among young people
What is the impact of vaccinating one hypothetical adolescent cohort compared with the current standard of care for gonorrhoea (antibiotics)? [126]	A decision-analysis model was populated using published healthcare utilization and cost data	Additional sensitivity analysis data is available for the economic impact (see section 7)	A two-dose adolescent vaccination campaign was assumed, with protective immunity starting at age 15 years and a base-case efficacy against gonorrhoea of 20% Duration of effect of 10 years	Adolescent vaccination with 4CMenB would prevent 83,167 (95% credible interval (CrI), 44,600–134,600) *N. gonorrhoeae* infections Excluding vaccination costs, direct medical costs for gonorrhoea would reduce by USD 28.7 million (95% CrI, $6.8-$70.0 million), and income and productivity losses would reduce by $40.0 million (95% CrI, $8.2-$91.7 million)
What is the vaccination’s potential impact and the feasibility of achieving the World Health Organization’s (WHO) target of reducing gonorrhoea incidence by 90% during 2018–2030? [127]	This study developed a stochastic transmission-dynamic model, incorporating asymptomatic and symptomatic infection and heterogeneous sexual practices in MSM Used data from England, which has a comprehensive, consistent, nationwide surveillance system	Using Markov chain Monte Carlo methods, the study fitted to gonorrhoea incidence in 2008–2017, then used Bayesian forecasting to examine an extensive range of scenarios. Accounted for uncertainty in estimated parameters by using 1000 samples from the joint posterior distribution	Vaccine coverage among eligible individuals was varied (50%−100%) for each strategy (i.e. “vaccination before entry” “vaccination on diagnosis” and “vaccination on attendance”)	Even in the worst-case scenario of untreatable infection emerging, the WHO target is achievable if all MSM attending sexual health clinics receive a vaccine offering ≥52% protection for ≥6 years. A vaccine conferring 31% protection (as estimated for MeNZB) for 2–4 years could reduce incidence in 2030 by 45% in the worst-case scenario and by 75% if >70% of resistant gonorrhoea remains treatable Even a partially-protective vaccine, delivered through a realistic targeting strategy, could substantially reduce gonorrhoea incidence, despite antibiotic resistance
What is the impact of vaccination on antimicrobial resistant *N. gonorrhoeae* among men who have sex with men (MSM)? [128]	Developed a compartmental model of *N. gonorrhoeae* transmission among MSM Estimated the impact of a partially protective vaccine (reducing susceptibility; 2-years protection) targeting high sexual activity MSM on AMR and prevalence until 2050.	Sensitivity analyses for varying levels of vaccine effectiveness and other modes of vaccine action Considered urogenital, anorectal and pharyngeal sites of infection together Development of AMR occurred in a multistage unidirectional process Conducted uncertainty analysis in which it was assumed that the resistant strain had a 5% lower transmission probability Calibration was done for unknown parameters that were unrelated to AMR development	Target population for this model was MSM 30% of positive cases are symptomatic Minimal inhibitory concentration distribution (MIC) overtime may result in the accumulation of genetic mutations that increase the MIC and result in AMR No fitness cost for each group of strains All positive cases were given ceftriaxone as empirical first-line treatment and with sufficient dosage to clear the gonococcal infection Of AMR cases treated with ceftriaxone, the fraction of men that will fail treatment will be higher for those infected with the reduced sensitivity strain Vaccine provided short protection, and MSM can be vaccinated multiple times	Vaccination against AMR *N. gonorrhoeae* among MSM provided 30% protection and immunity lasting two years However, a vaccine of 30% vaccine effectiveness does not eradicate AMR but instead slows down the process of development of AMR at all levels of vaccine uptake If there is a vaccine uptake of 40%, a vaccine needs to provide 90% protection to eradicate AMR
What is the vaccination’s impact on the incidence of *N. gonorrhoeae* infection among MSM? [129]	Developed a mathematical model of 10,000 MSM. The model was simulated for 30 years with vaccination introduced 20 years after the initiation of gonorrhoea transmission in year 0.	Each individual has three possible anatomical sites of gonococcal infection (urethra, anorectum, oropharynx), and bidirectional transmission can occur between any two of the three sites. Three types of vaccine mode of actions were investigated, vaccine efficacy against acquiring gonorrhoea, vaccine efficacy in reducing the probability of transmission, and vaccine efficacy in reducing symptoms in a vaccinated infected individual.	Infection at any anatomical site is localised and independent from infection at another site. Treatment would clear infections at all sites simultaneously. 80% of MSM would receive routine STI testing annually. 30% of MSM presenting for routine STI testing would receive the vaccination. Individuals of the model receive only one dose of gonococcal vaccine in their lifetime, and vaccine-conferred protection would start to wane after 2 years. Approximately 40% of the total population would be vaccinated against gonorrhoea 2 years after vaccination.	There will be a 94% relative reduction in the prevalence of gonorrhoea 2 years after the introduction of vaccination, and elimination within 5 years, given an available gonococcal vaccine that confers 100% protection against acquiring gonorrhoea. A vaccine that confers 100% transmission suppression efficacy would still result in a 90% decrease in gonorrhoea prevalence 2 years after the introduction of vaccination. Even with vaccines that confer lower protective or transmission efficacy, there would still be a substantial impact on disease prevalence. With vaccines that confer < 50% protective and transmission suppression efficacy, as long as a booster shot is available every 3 years on average, elimination is possible within 8 years. Vaccine’s impact on prevalence may be reduced if not effective against infection at all anatomical sites.
What is the vaccination’s impact on the incidence of *N. gonorrhoeae* infection among MSM? [129]	Developed a mathematical model of 10,000 MSM. The model was simulated for 30 years with vaccination introduced 20 years after the initiation of gonorrhoea transmission in year 0.	Each individual has three possible anatomical sites of gonococcal infection (urethra, anorectum, oropharynx), and bidirectional transmission can occur between any two of the three sites. Three types of vaccine mode of actions were investigated, vaccine efficacy against acquiring gonorrhoea, vaccine efficacy in reducing the probability of transmission, and vaccine efficacy in reducing symptoms in a vaccinated infected individual.	Infection at any anatomical site is localised and independent from infection at another site. Treatment would clear infections at all sites simultaneously. 80% of MSM would receive routine STI testing annually. 30% of MSM presenting for routine STI testing would receive the vaccination. Individuals of the model receive only one dose of gonococcal vaccine in their lifetime, and vaccine-conferred protection would start to wane after 2 years.	There will be a 94% relative reduction in the prevalence of gonorrhoea 2 years after the introduction of vaccination, and elimination within 5 years, given an available gonococcal vaccine that confers 100% protection against acquiring gonorrhoea. A vaccine that confers 100% transmission suppression efficacy would still result in a 90% decrease in gonorrhoea prevalence 2 years after the introduction of vaccination. Even with vaccines that confer lower protective or transmission efficacy, there would still be a substantial impact on disease prevalence.
			Approximately 40% of the total population would be vaccinated against gonorrhoea 2 years after vaccination.	With vaccines that confer < 50% protective and transmission suppression efficacy, as long as a booster shot is available every 3 years on average, elimination is possible within 8 years. Vaccine’s impact on prevalence may be reduced if not effective against infection at all anatomical sites.
What is the cost-effectiveness of the gonococcal vaccine for MSM in England? [130]	Integrated transmission-dynamic health-economic model, calibrated using Bayesian methods to surveillance data (from the Genitourinary Medicine Clinic Activity Dataset and the Gonococcal Resistance to Antimicrobials Surveillance Programme) on men who have sex with men (MSM) in England	Considered vaccination of MSM from the perspective of sexual health clinics, with and without vaccination offered to all adolescents in schools (vaccination before entry [VbE]), comparing three realistic approaches to targeting, vaccination on attendance (VoA) for testing; vaccination on diagnosis (VoD) with gonorrhoea; or vaccination according to risk (VaR), offered to patients diagnosed with gonorrhoea plus individuals who test negative but report having more than five sexual partners per year.	Simulated introduction of the gonococcal vaccine in 2022 using vaccines varying in efficacy (protection against acquiring infection 1–100%) and duration of protection (1–20 years), with four different targeting strategies (VbE, VoA, VoD, VaR).	VbE has little impact on gonorrhoea diagnoses, with only 1·7% of MSM vaccinated per year. VoA has the largest impact but requires more vaccine doses than any other strategy, whereas VoD has a moderate impact but requires many fewer doses than VoA VaR has almost the same impact as VoA but with fewer doses administered than VoA. VaR is the most cost-effective strategy for vaccines of moderate efficacy or duration of protection (or both), although VoD is more cost-effective for very protective and long-lasting vaccines. A hypothetical gonorrhoea vaccine’s value is increased more by improving its efficacy than its duration of protection—e.g., 30% protection lasting 2 years has a median value of £48 (22–85) per dose over 10 years; doubling efficacy increases the value to £102 (53–144) whereas doubling the duration of protection increases it to £72 (34–120).

**Table 8:** Overview of modelling studies on gonococcal vaccines that measure anticipated socio-economic impact of the vaccine.

Policy question	Assessment method/measure	Additional information specific to models	Assumptions	Outcomes/ interpretation
Potential health and economic benefits of combating antibiotic-resistant gonorrhoea in the United States[131]	Impact on AMR	Two scenarios were modelled: (1) The prevalence of cephalosporin-resistant gonorrhoea remains at 2% (2) The prevalence of cephalosporin-resistant gonorrhoea increased linearly from 2% at the start to 15% after six years and then at 15% until year 10.	Emergence of cephalosporin-resistant gonorrhoea would have a similar impact on gonorrhoea incidence to the emergence of ciprofloxacin resistance in the late 1990s. Gonorrhoea incidence rate would remain constant if the percentage of cephalosporin-resistant gonococcal infection did not change. The annual number of gonococcal infections in the United States is 820,000 without emerging resistance. Lifetime cost per gonococcal infection in males assumed as US$86, and in females, US$383.	Compared to scenario 1, gonorrhoea rates in scenario 2 were estimated to be 2% higher in year 1, 14% higher in year 5, and 22% higher in year 10, which would result in an additional 1.2 million cases in a 10-year period, of which 579 would lead to gonorrhoea-attributable HIV infections. Additional cost in scenario 2 was estimated to be $378.2 million, which includes $170.5 million for treating gonorrhoea-attributable HIV infections Significant health and economic losses can be avoided by maintaining the prevalence of ceftriaxone-resistant *N. gonorrhoeae* to lower than 2%. The cost of implementing strategies to prevent emerging AMR, such as introducing a vaccine, can at least be partially offset by averting the costs of emerging resistance.
To compare the impact of possible gonorrhoea vaccination with the current standard of treatment with antibiotics; the economically justifiable price of potential gonorrhoea vaccines [126]	Cost-effectiveness analysis / QALYs	Decision-analytic model Sensitivity analyses revealed that most costs and QALYs gained by the vaccination program were achieved through co-reduction of gonorrhoea-attributable HIV infections. The vaccine’s cost-effectiveness was affected by the efficacy of available antibiotic treatment. Higher antibiotic efficacy would result in lower cost-effectiveness for vaccination. If the cost of new antibiotic treatment was higher than today’s available option, then vaccination would be more cost-effective.	Vaccine efficacy of 20% against gonorrhoea with an average duration of effect of 10 years. A theoretical cohort consisting of 2,047,000 men and 1,957,000 women aged 15 years old was used in this model. Vaccination rate of 75%. The same vaccination rate was assumed for men and women and at-risk populations. This model has a reinfection rate of 4.5% per disease cycle. For each case of *N. gonorrhoeae* infection prevented through the vaccination program, an additional 0.5 cases would be prevented in the general population. 97% efficacy of antibiotics for treating gonorrhoea.	The model predicts that for the given vaccine efficacy and timeframe, 83,617 *N. gonorrhoeae* infections (disregarding first infection or reinfection) can be prevented within the theoretical cohort over a lifetime. Without vaccination, the model predicts 844,000 *N. gonorrhoeae* infections within this cohort. 1,265 quality-adjusted life-years (QALYs) could be saved by vaccinating the theoretical cohort. Without vaccination, the model predicts a loss of 14,106 QALYs within this cohort. Direct medical cost decreases by $28.7 million from $56 million in the unvaccinated scenario. Income and productivity losses would reduce to $40.0 million from $75 million per unvaccinated cohort. Even at low effectiveness of just 20%, the gonococcal vaccine could still substantially reduce the disease burden and cost to justify a price of $26.10 per dose.

**Table 9:** Overview of expectations of evidence that are likely to be required to support a global / regional / national policy recommendation or financing for gonococcal vaccines.

Parameter for policy/financing consideration	Assumptions/considerations
Vaccine safety and efficacy	Vaccines will need to be shown safe and efficacious in clinical trials [7].
Vaccine impact on antibiotic use and AMR	Demonstrating vaccine impact in reducing antibiotic prescribing and/or AMR-strains in post-introduction observational studies would facilitate policy and financing decisions [97,128,131].
Global, regional, and (where feasible) national gonorrhoea-associated disease burden data	Global and regional infection estimates exist; however, disease burden estimates will be important in influencing policy and financing discussions [1].
Global, regional, and (where feasible) national data on gonococcal AMR	Global AMR surveillance is ongoing but could be expanded to reach more countries, particularly LMICS. Increasing AMR could greatly influence policy decisions [67].
Favourable cost-effectiveness	Several modelling studies have demonstrated the potential impact of gonococcal vaccines. however, further work is needed on cost-effectiveness in different epidemiologic scenarios. Countries will be more likely to take up vaccines if cost-effectiveness analyses show favourable value for money [126,130].
Product price acceptable to Gavi investment case for use in Gavi-eligible countries	LMICs that are Gavi eligible will apply for the use of gonococcal vaccines only if Gavi support is available.

**Table 10:** Access and implementation feasibility assessment for gonoccocal vaccines and MenB vaccines with potential cross-protection.

	Intervention	
	Gonococcal vaccines	MenB vaccines with potential cross-protection

Possibility of implementation within existing delivery systems	High to very high (young adolescents)	High to very high (young adolescents)
	Low to moderate (older young people and populations at higher risk)	Low to moderate (older young people and populations at higher risk)
	Vaccines targeted to young adolescents before their sexual debut could be implemented with existing adolescent vaccination programs, including school-based programs, e.g., with the HPV vaccine. Targeting this population without needing boosters before the period of highest risk will require a sufficient duration of protection. No vaccine delivery systems currently exist for other target populations for gonococcal vaccines; however, a variety of sexual health service platforms, e.g., PrEP clinics for HIV prevention, could be leveraged for vaccination programs. Experience with COVID-19 vaccine implementation might also improve reaching these groups.	The same considerations according to target populations apply for MenB vaccines as for standalone gonococcal vaccines. However, in settings where MenB vaccines are already recommended and delivered in overlapping target groups for gonococcal infection, incorporation into existing programs would be more easily facilitated.

Commercial attractiveness	Low	Moderate to high
	Both HIC and LMIC targets exist; however, target populations in HICs are typically small (key populations). Target populations in LMICs tend to be larger (e.g., some countries have high general population prevalence); however, the burden of infection and disease is not well defined in most LMIC settings. Gavi support will depend on cost-effectiveness in different settings. Increasing AMR would increase commercial attractiveness in all settings.	Gaining an additional indication for an existing vaccine would only increase its potential for use and cost-effectiveness in different settings. The burden and overlap between MenB disease and gonococcal infection have not been well defined in all settings. In addition, target populations may not overlap. Increasing AMR would increase commercial attractiveness in all settings.

Clarity of licensure and policy decision pathway	Low to moderate	Moderate to high
	The licensure pathway is relatively clear, although an infection indication is preferred over a disease indication for various reasons, e.g., the role of asymptomatic infections in propagating AMR and difficulties in measuring upper genital tract disease in clinical trials. Regulators often prefer disease indications, although not uniformly. Policy decisions may vary greatly in HICs vs LMICs and among countries in both settings, based on several factors, including epidemiology and cost-effectiveness, as well as AMR.	Given that MenB vaccines are already licensed for another condition, a lower efficacy could be acceptable for broadening the use of MenB vaccines to prevent gonococcal infection compared with the use of a standalone gonococcal vaccine, particularly for settings with a history of MenB endemic disease and/or outbreaks.

Expected financing mechanism	Low to moderate	Moderate
	Interest from global funders, including Gavi, is unclear and will likely depend on data related to disease burden, cost-effectiveness, and importantly, AMR. There is limited country-level data on gonococcal epidemiology and disease burden to inform decision-making by national procurement agencies once the vaccines are available.	Cost-effectiveness, and therefore interest from global funders, would likely increase with expanding indications for MenB vaccines. Again, there is limited data on country-level disease burden and epidemiology, and overlap of the two conditions, which will be required for decision-making by national procurement agencies.

Ease of uptake	Moderate	High
	For adolescents, a well-defined target population exists, as for HPV vaccines. However, in many settings, vaccine hesitancy has been an issue for HPV vaccines, which are widely seen as cancer-prevention vaccines; gonococcal vaccines will likely be more clearly associated with a sexually transmitted infection, which may affect acceptability, particularly to parents of adolescents.	The likelihood of acceptability is higher for MenB vaccines that have additional efficacy against gonorrhoea. Meningitis may be perceived as less stigmatizing than gonococcal infection.
	Acceptability is likely higher for specific populations at greater risk for gonorrhoea; however, these populations may not be as well defined or as easy to access (e.g., MSM in LMICs).	

**Table 11:** Knowledge gaps and data and research needs for the development of gonoccocal vaccines.

Area	Data gap or research need	Notes and additional considerations
*Obtaining better epidemiologic data regarding Neisseria gonorrhoeae (Ng) infection, disease, antimicrobial resistance (AMR), and natural history*		
Infection	• Improve global and regional estimates of Ng infection • Obtain prevalence and incidence data regarding Ng infection from more settings and populations • Evaluate overlap of Ng infection epidemiology with that of meningococcal serogroup B infection and introduction of meningococcal B vaccines across countries	• Development and validation of cheap, feasible Ng diagnostic tests is crucial for obtaining better data, especially for low- and middle-income countries (LMICs) • Conduct strategically determined, additional prevalence studies in selected LMIC areas where most data are being imputed • The World Health Organization (WHO) has published a standard protocol for conducting gonorrhoea prevalence surveys in antenatal settings • Use of existing Ng infection data from clinical trials (e.g., HIV prevention, human papillomavirus (HPV) vaccine, maternal studies) should be explored
Disease	• Improve global and regional estimates of Ng-associated clinical and disease outcomes • Obtain prevalence and incidence data regarding Ng-associated disease from more settings and populations, particularly in LMICs • Determine attributable fractions of such outcomes as PID and tubal factor infertility (TFI) caused by gonorrhoea	• Outcomes can include urethral discharge, pelvic inflammatory disease (PID), infertility, ectopic pregnancy, chronic pelvic pain, adverse birth outcomes, ophthalmia neonatorum and other eye disease, Ng-associated HIV infection, and other outcomes (e.g., epididymitis, proctitis, disseminated gonococcal infection, or male infertility) • Systematic reviews can be conducted first to summarize what is currently known about overall disease burden (e.g., all infertility or all TFI), about Ng-associated disease burden (e.g., Ng-associated PID and infertility), and about the methods used for attributing etiology to Ng (e.g., Ng serologic tests) • Updated etiologic studies of PID using cervical testing and of the proportion of infertility that is tubal factor, are needed in diverse settings • Improved methods for measuring upper-genital tract disease (e.g., biomarkers, radiology, or case definitions) and for measuring past Ng infection (e.g., improved serologic tests) would be valuable • Explore potential data sources regarding gonorrhoea-associated adverse pregnancy outcomes
AMR	• Obtain more globally representative assessments of Ng AMR, transmission, and clinical treatment failures • Determine trends in Ng AMR in more settings and among more populations	• Increase number of countries testing gonococcal isolates for AMR and reporting through WHO’s Gonococcal Antimicrobial Surveillance Programme (GASP) • Improved diagnostic tests, including rapid tests for Ng AMR would aid evaluation • Systems are needed for identifying and reporting clinical treatment failures for gonorrhoea and other key AMR metrics (e.g., sentinel surveillance sites in LMIC settings)
Natural history and transmission	• Improve understanding of the proportion, predictors, and timing of Ng cervical infections ascending to the upper-genital tract, causing PID and resulting in long-term sequelae • Gain insight into the factors associated with acquisition, transmission, and duration of infection, including at multiple anatomical sites	• Innovative, ethical study designs are needed for assessing the natural history of Ng infection • Explore study designs used for understanding chlamydia natural history and their role in evaluating gonorrhoea (e.g., evaluating rates of PID in the interval between testing and treatment or using serial specimens from existing prospective studies) • Couples studies might help researchers understand transmission from different anatomical sites and other factors like bacterial load
*Modelling gonococcal infection, disease, AMR, economic burden, and theoretical vaccine impact and cost-effectiveness*		
Overall	• Review and summarize the models that have been published, are ongoing, or are planned • Determine priority data needs for robust modelling • Prioritize and coordinate modelling efforts across groups, initiatives, and interventions	• Modelling efforts related to gonorrhoea across different initiatives (e.g., AMR) and interventions (e.g., new antibiotic development) will need to be coordinated to increase model efficiency and utility • Strengthened data on burden ofinfection, disease, AMR, transmission, and natural history is important for all models • Consensus regarding a plausible range of important model assumptions will be valuable • Model comparisons can strengthen robustness of conclusions from modelling studies
Models of Ng infection, disease, AMR, and economic burden	• Develop dynamic models of Ng infection, disease, and AMR in varied settings • Estimate global and regional economic burden of Ng infection and disease • Predict future trends in Ng infection, disease, AMR, and costs	• Improve data for modelling inputs and assumptions, including information about key populations and sexual networks • Modelling can be difficult for low prevalence infections with heterogenous distribution within the population • Predictions of the effect of increasing AMR on infection and disease incidence and on costs can help refine the value of a vaccine
Vaccine impact models	• Model the potential effectiveness of a future Ng vaccine in the context of the observed epidemiology and disease burden in different settings • Model potential vaccine impact against different assumptions and scenarios	• How to value AMR, a vaccine’s potential effect on AMR, and a vaccine’s impact in the context of AMR will be key to understanding overall vaccine impact and value • Understanding vaccine impact considering different target populations, immunization strategies, and efficacies can guide PPCs in addition to value propositions • Models should consider both high-income countries (HICs) and LMIC settings • Will be important to include alternative interventions (e.g., new antibiotics) in models
Cost-effectiveness models	• Model the potential cost-effectiveness of a future Ng vaccine given the observed and predicted health and economic burden in different settings • Model potential cost-effectiveness against different assumptions and scenarios	• Systematic reviews can assess what is known about gonorrhoea health care-seeking and -usage, and costs of care and treatment, for both infection and disease • Work will be needed to refine estimates of disability adjusted life-years and quality adjusted life-years considering all Ng outcomes • Cost-effectiveness analyses can guide preferred product characteristics (PPCs) in addition to value assessments
*Advancing basic science, translational, immunobiologic, and clinical research*		
Experimental systems	• Refine animal models and *in vitro* systems to expand the range of features of human infections that can be studied and to enable vaccine candidate assessment • Explore and optimize use of controlled human infection models (CHIMs) to study Ng immune responses and vaccine efficacy	• Continued understanding of complex Ng immunobiology can lead to new target antigens, novel adjuvant, or delivery systems • Improved preclinical systems for evaluating vaccine candidates can facilitate entry into clinical evaluation • Better data about pathogenesis and immunity in human infections can be compared with animal models to refine them • Current CHIMs exist only for male urethral infection, but could be developed for others (e.g., postmenopausal women)
Antigen discovery and vaccinology	• Continue to take advantage of new technologies to screen for new antigenic targets and define the most promising list of candidates • Further define mechanisms of immunity and immune evasion, which could help in developing adjuvants and delivery platforms for Ng vaccines • Evaluate vaccine candidates in preclinical models or CHIMs	• Evaluation of vaccine candidates is challenging, given that no established surrogate markers or correlates of protection against Ng exist • Given antigenic variability of Ng, a combination of antigens might be needed to provide broad protection against different Ng strains • Findings related to meningococcal outer membrane vesicle (OMV) vaccines and possible cross-protection for Ng can provide direction for vaccine development
Translational, immunobiologic, and clinical studies	• Conduct studies to obtain better data on human immune responses to Ng infection, including prospective evaluations of responses associated with reinfection • Evaluate the effect of licensed meningococcal serogroup B OMV vaccines on Ng acquisition • Evaluate the effect of coinfections, the microbiome, and hormonal status on Ng infection, disease, and immune response • Better understand the role of oropharyngeal and rectal infection in Ng transmission and promotion of AMR • Facilitate progression of promising preclinical candidates into clinical evaluation as soon as possible	• Clinical studies are needed to examine host factors and immune responses during Ng infection, and those predicting the likelihood of infection, reinfection, or ascension to the upper-genital tract • Innovative studies can be modelled on those conducted for chlamydia (e.g., studies reporting that clearance of infection between testing and treatment is linked with reduced risk for repeat infection) • People with an increased risk for complicated Ng disease from complement disorders can provide clues to correlates of risk and protection • Efficacy of meningococcal serogroup B vaccines ideally will be evaluated through clinical trials specifically designed for examining efficacy against Ng infection or disease acquisition; prospective observational studies as the vaccines are rolled out in new areas can also add insight • Couples studies might be useful for evaluating transmission and factors associated with transmission • Correlates of protection might be difficult to identify but would be highly useful; although not essential for vaccine development and licensing, they can make bridging studies easier
*Encouraging investment and planning for policy and implementation decisions in advance*		
Value assessment and PPCs	• Consolidate data on burden of disease, economic burden, and vaccine impact and cost-effectiveness • Understand drivers of gonococcal vaccine development and who the main stakeholders are • Obtain country-level input regarding the potential value of Ng vaccines and features of a vaccine that would be essential and those that would be desirable in different settings	• Improving the quantity and quality of underlying disease data is crucial for developing the vaccine value assessment and PPCs • Considering the interests and needs of different stakeholders is vital (e.g., WHO’s Strategic Advisory Group of Experts, Gavi, the Vaccine Alliance, funders, vaccine developers, national policymakers, health care providers, individuals at risk, parents, and civil society) • Value-of-vaccines assessments consider more than just health benefits and can also include broader societal and public value
Acceptability and implementation	• Evaluate the level of awareness and knowledge about gonorrhoea and Ng AMR and risk perception among young people and their parents, health care providers, and policymakers • Obtain country-level information regarding the likely acceptability of and demand for Ng vaccines from a broad range of countries • Evaluate the potential acceptability of Ng vaccines and potential barriers to acceptance or uptake by individuals and communities for different populations and settings • Assess the potential delivery systems for gonococcal vaccines for different target populations in varied health care systems	• Increasing awareness of Ng AMR and treatment failures can affect both awareness and demand for Ng vaccines • Understanding potential acceptability of Ng vaccines and how they would be used, including country-level input, early in vaccine development can help guide development of vaccines that are suited for global use and able to be implemented more quickly upon licensure

## Data Availability

No data was used for the research described in the article.
